# Comparative genomics analysis to explore the biodiversity and mining novel target genes of *Listeria monocytogenes* strains from different regions

**DOI:** 10.3389/fmicb.2024.1424868

**Published:** 2024-06-19

**Authors:** Bo Zhang, Honglin Ren, Xiaoxu Wang, Cheng Han, Yuanyuan Jin, Xueyu Hu, Ruoran Shi, Chengwei Li, Yuzhu Wang, Yansong Li, Shiying Lu, Zengshan Liu, Pan Hu

**Affiliations:** ^1^State Key Laboratory for Diagnosis and Treatment of Severe Zoonotic Infectious Diseases, Key Laboratory for Zoonosis Research of the Ministry of Education, Institute of Zoonosis, and College of Veterinary Medicine, Jilin University, Changchun, China; ^2^Institute of Special Animal and Plant Sciences, Chinese Academy of Agricultural Sciences, Changchun, Jilin, China

**Keywords:** *Listeria monocytogenes*, comparative genomics, pan-genomics, biodiversity, target genes

## Abstract

As a common foodborne pathogen, infection with *L. monocytogenes* poses a significant threat to human life and health. The objective of this study was to employ comparative genomics to unveil the biodiversity and evolutionary characteristics of *L. monocytogenes* strains from different regions, screening for potential target genes and mining novel target genes, thus providing significant reference value for the specific molecular detection and therapeutic targets of *L. monocytogenes* strains. Pan-genomic analysis revealed that *L. monocytogenes* from different regions have open genomes, providing a solid genetic basis for adaptation to different environments. These strains contain numerous virulence genes that contribute to their high pathogenicity. They also exhibit relatively high resistance to phosphonic acid, glycopeptide, lincosamide, and peptide antibiotics. The results of mobile genetic elements indicate that, despite being located in different geographical locations, there is a certain degree of similarity in bacterial genome evolution and adaptation to specific environmental pressures. The potential target genes identified through pan-genomics are primarily associated with the fundamental life activities and infection invasion of *L. monocytogenes*, including known targets such as *inlB*, which can be utilized for molecular detection and therapeutic purposes. After screening a large number of potential target genes, we further screened them using hub gene selection methods to mining novel target genes. The present study employed eight different hub gene screening methods, ultimately identifying ten highly connected hub genes (*bglF_1, davD, menE_1, tilS, dapX, iolC, gshAB, cysG, trpA, and hisC*), which play crucial roles in the pathogenesis of *L. monocytogenes*. The results of pan-genomic analysis showed that *L. monocytogenes* from different regions exhibit high similarity in bacterial genome evolution. The PCR results demonstrated the excellent specificity of the *bglF_1* and *davD* genes for *L. monocytogenes*. Therefore, the *bglF_1* and *davD* genes hold promise as specific molecular detection and therapeutic targets for *L. monocytogenes* strains from different regions.

## Introduction

*Listeria monocytogenes*, a foodborne pathogen, is a Gram-positive rod-shaped bacterium belonging to the genus *Listeria* within the phylum Firmicutes et Bacillota. It is a facultative anaerobe (Wang et al., 2021; Lourenco et al., 2022). Currently, there are 30 species of *Listeria* registered in the prokaryotic nomenclature database LPSN (last accessed on November 7, 2023) (Lu et al., 2022). Among the *Listeria* genus, *L. monocytogenes* is commonly considered a pathogenic strain and the most prevalent species (Sheng et al., 2017; Cain et al., 2023). It can cause listeriosis in humans, particularly in immunocompromised individuals such as neonates, elderly, pregnant women, and those with weakened immune systems, and can present with various symptoms including mild diarrhea, meningitis, and sepsis (Locatelli et al., 2017; Radoshevich and Cossart, 2018). The pathogenicity of bacteria and their unique ability to adapt to their habitats have a distinctive genetic basis. Numerous relationships between genes and pathogenic phenotypes have been identified and studied in *L. monocytogenes* (Fox et al., 2016; Disson et al., 2021). However, the genetic basis of *L. monocytogenes* pathogenicity and environmental adaptability is not fully understood and requires further elucidation.

In recent years, with the widespread application of next-generation sequencing and third-generation sequencing technologies, a large number of *L. monocytogenes* genomes have been sequenced and shared (Jordan and McAuliffe, 2018). Comparative genomics analysis of *L. monocytogenes* strains in different regions can deepen our understanding of their genetic mechanisms for adapting to different environments and their pathogenic lifestyles (Lomonaco et al., 2015). The objective of this study was to employ comparative genomics to unveil the biodiversity and evolutionary characteristics of *L. monocytogenes* strains from different regions, screening for potential target genes and mining novel target genes, thus providing significant reference value for the specific molecular detection and therapeutic targets of *L. monocytogenes* strains. Therefore, we conducted comparative genomics research on *L. monocytogenes* strains from different regions including America, Europe, and Asia. The pan-genomes, core genomes, and potential target genes of each *L. monocytogenes* strain were analyzed, and each strain was subjected to multilocus sequence typing (MLST). Functional analysis of the potential target genes in *L. monocytogenes* was conducted using GO and KEGG annotations. Furthermore, a protein-protein interaction (PPI) network was constructed for potential target genes of *L. monocytogenes*, and eight different hub gene analysis methods were utilized to screen novel target genes from the potential target genes. Finally, the virulence genes, antibiotic resistance genes, plasmids, prophages, and CRISPR-Cas systems of each *L. monocytogenes* strain were investigated.

## Materials and methods

### Data retrieval and management

In this study, a total of 355 genome sequences were retrieved and downloaded from the NCBI genome database (last accessed on November 7, 2023), including 343 *L. monocytogenes* strains from three different regions (223 from America, 91 from Europe, and 29 from Asia), as well as 12 other *Listeria* species and *non-Listeria* bacterial genomes. Detailed information of the studied *L. monocytogenes* genomes, such as GenBank accession numbers, strain names, genome size, GC content, number of contigs and N50, are summarized in Supplementary Tables S1, S2 (Palma et al., 2017; Chiaverini et al., 2021). To ensure greater representativeness, *L. monocytogenes* isolated from cerebrospinal fluid were prioritized, as these strains can induce severe clinical manifestations. In brief, this entails downloading all genomes of *L. monocytogenes* strains isolated from cerebrospinal fluid in NCBI databases pertaining to America, Europe, and Asia for subsequent analysis. To ensure the specificity of the target genes obtained, *non-Listeria* bacterial genomes were selected based on their high coverage and homology with *Listeria* sequences, using Gram-positive reference strains for analysis. Typically, bacteria belonging to the Gram-positive rods exhibit a genomic coverage and homology percentage exceeding 95% (Wang et al., 2022).

### Pan-genomic analysis of *Listeria monocytogenes* and non-target bacterial strains from different regions

The analysis of pan-genomic comparison of *L. monocytogenes* and non-target strains can be used to screen potential target genes. The potential target genes refer to those genes that are unique to *L. monocytogenes* strains and are absent in non-target strains (Li et al., 2021a). In brief, all analyzed genome sequences were annotated using Prokka v1.14.6 (Seemann, 2014), and the output results of Prokka were used for pan-genomic analysis with Roary v3.11.2 (Page et al., 2015). A core genome was determined for each isolate using a 99% cutoff, with a BLASTP identity cutoff of 85% (Pang et al., 2019). Genes that matched with all *L. monocytogenes* strains genome sequences were considered highly conserved and used for subsequent comparisons with other *Listeria* species and *non-Listeria* bacterial genomes.

Pan-genome clusters were defined as core-genes: present in all isolates; soft-core genes: present in at least 95% of isolates; shell-genes (accessory genes): present between 15 and 95% of isolates; and cloud-genes (unique genes): present in less than 15% of isolates (Mafuna et al., 2022).

The potential target genes were screened according to the following criteria: 100% presence in *L. monocytogenes* strains and no presence in non-target bacterial strains. Then, these potential target genes were used screened against the nucleotide collection (nr/nt) databases using the online BLAST program to ensure specificity (Li et al., 2021b).

### Multilocus sequence typing analysis

*L. monocytogenes* was subjected to MLST using 7 housekeeping genes (*abcZ*, *bglA*, *cat*, *dapE*, *dat*, *ldh*, and *lhkA*) as markers (Henri et al., 2016). MLST profiles were obtained from the *Listeria* database hosted by the Pasteur Institute, France. The MLST v.2.18.0 was used to align reads against these profiles to determine the sequence types (STs), Clonal Complex values (CC) and lineage for each genome (Mafuna et al., 2022).

### Phylogenetic analysis

To investigate the phylogenetic relationships between the 343 *L. monocytogenes* from different regions, all the core single-copy genes were extracted and aligned using MAFFT v7.490 (Lu et al., 2022). Then, the aligned sequences were concatenated for each strain with a uniform gene order, and GBLOCKS 0.91b was utilized to remove the poorly aligned positions and divergent regions (Lu et al., 2022). MEGA 11 was used to compute the maximum likelihood (ML) phylogenetic tree (Lu et al., 2022). The online tool Interactive Tree of Life (iTOL) v6 was used to visualize the tree with midpoint rooting, and the geographic location, and the ST typing of each strain were annotated on the tree (Lu et al., 2022).

### Functional characteristics of potential target genes

In order to investigate the functional characteristics of genes present exclusively in *L. monocytogenes* strains and absent in non-target bacterial strains (potential target genes), annotation analysis was performed using Gene Ontology enrichment analysis (GO analysis) and Kyoto Encyclopedia of Genes and Genomes enrichment analysis (KEGG analysis) (Gao et al., 2021), and the results were integrated.

### Protein-protein interaction network analysis and identification of novel target genes

In this study, the STRING database was utilized to construct PPI networks, and these networks were visualized using Cytoscape v3.10.1 (Li et al., 2021c). The CytoHubba function in Cytoscape v3.10.1 was employed to identify hub genes (novel target genes) from the PPI. The CytoHubba function employs eight distinct algorithms to rank genes in the PPI network, which include Degree, Betweenness, BottleNeck, Closeness, Edge Percolated Component (EPC), Maximum Neighborhood Component (MNC), Radiality, and Stress. The top 10 genes with the highest scores are selected as hub genes (Zhang et al., 2019).

### Prediction of virulence factors and antibiotic resistance genes of *Listeria monocytogenes*

The prediction of virulence factor-related genes and antibiotic resistance genes in the *L. monocytogenes* genome was conducted to determine their presence. The Virulence Factors of Pathogenic Bacteria (VFDB) database and The Comprehensive Antibiotic Resistance (CARD) database were employed to detect virulence genes and antibiotic resistance genes in the *L. monocytogenes* genome (Tan et al., 2015; Mafuna et al., 2021), and the results are summarized and presented in a heatmap.

### Prediction of MGEs of *Listeria monocytogenes*

Mobile Genetic Elements (MGEs) refer to a class of genetic elements capable of spreading or transferring within a genome, such as plasmids and prophages, which can facilitate the evolution of microorganisms (Castro et al., 2021). The plasmid database PLSDB and the PHAge Search Tool-Enhanced Release (PHASTER) prophage database were utilized to detect MGEs in the *L. monocytogenes* genome (Bosi et al., 2017; Matle et al., 2019). The detection results were summarized and presented in a heatmap.

### Prediction of CRISPR-Cas systems of *Listeria monocytogenes*

Predict the genome of *L. monocytogenes* to determine the presence of the CRISPR-Cas system. CRISPRCasFinder was used for the detection and typing of the Clustered Regularly Interspaced Short Palindromic Repeats and Cas genes (CRISPR-Cas) system of the *L. monocytogenes* (Parsons et al., 2021). To obtain the functional CRISPR-Cas system, the presence of both the CRISPR sequence and Cas genes was considered as evidence for an actual CRISPR-Cas system and used for the further analysis, and the detection results are summarized and displayed in a heatmap.

### Specific primer design and PCR detection conditions for *Listeria monocytogenes*

Primer design for the sequences of *bglF_1* and *davD* genes was performed using Primer Premier 5 software (Table 1) (He et al., 2022). The primers were synthesized by Sangon Biotech Co., Ltd., Shanghai, China. Primer specificity was tested by PCR analysis of strains from the laboratory collection. Total reaction volume was 25 μL, including 12.5 μL of 2 × Es Taq MasterMix (CWBIO, Beijing, China), 1 μL each of forward and reverse primers (10 μM), 8.5 μL of sterile water, and 2 μL of the purified bacterial genomic DNA as a template. An equal volume of sterile distilled water was used instead of the template as a negative control. PCR thermal cycling involved an initial denaturation step at 95°C for 10 min, followed by 35 cycles of denaturation at 95°C for 30 s, annealing at 56°C for 30 s, and elongation at 72°C for 1 min, with a final elongation at 72°C for 10 min. PCR products were evaluated by 2% agarose electrophoresis.

**Table 1:** Specific target genes and primers used for the detection of *L. monocytogenes*.

Gene	Sequence length/bp	Primer	Sequence (5′/3′)	Encoded protein	Product size/bp
bglF_1	1857	bglF_1F	AAGTGGCTGTCATGTTCG	PTS system beta-glucoside-specific EIIBCA component	616
		bglF_1R	ATCGCTACTCCTGCTCCC		
davD	1,467	davDF	AGTTGCGGCCATTACTCC	Glutarate-semialdehyde dehydrogenase	567
		davDR	TTGTCAATCGCATCTTCG		

## Results

### Genome statistics and general features

By querying the NCBI genomic database, we identified 343 strains of *L. monocytogenes*. We downloaded and curated the whole-genome sequences of these *L. monocytogenes* strains from the NCBI genomic database, along with the corresponding information (Supplementary Tables S1, S2). Among them, there were 223 isolates from America, with an average genome size of 2.98 (2.8–3.2) Mbp, an average GC content of 37.98 (37.5–38.0)%, the number of contigs ≤214 and an average N50 of 334,248. In Europe, there were 91 *L. monocytogenes* isolates, with an average genome size of 2.96 (2.9–3.2) Mbp, an average GC content of 37.98 (37.5–38.0)%, the number of contigs ≤42 and an average N50 of 656,340. In Asia, there were 29 *L. monocytogenes* isolates, among which, one possesses a complete genome, with an average genome size of 2.96 (2.8–3.1) Mbp, an average GC content of 38%, the number of contigs ≤72 and an average N50 of 699,124.

### Pan-genomic analysis of *Listeria monocytogenes* strains in different regions

**Figure 1:**
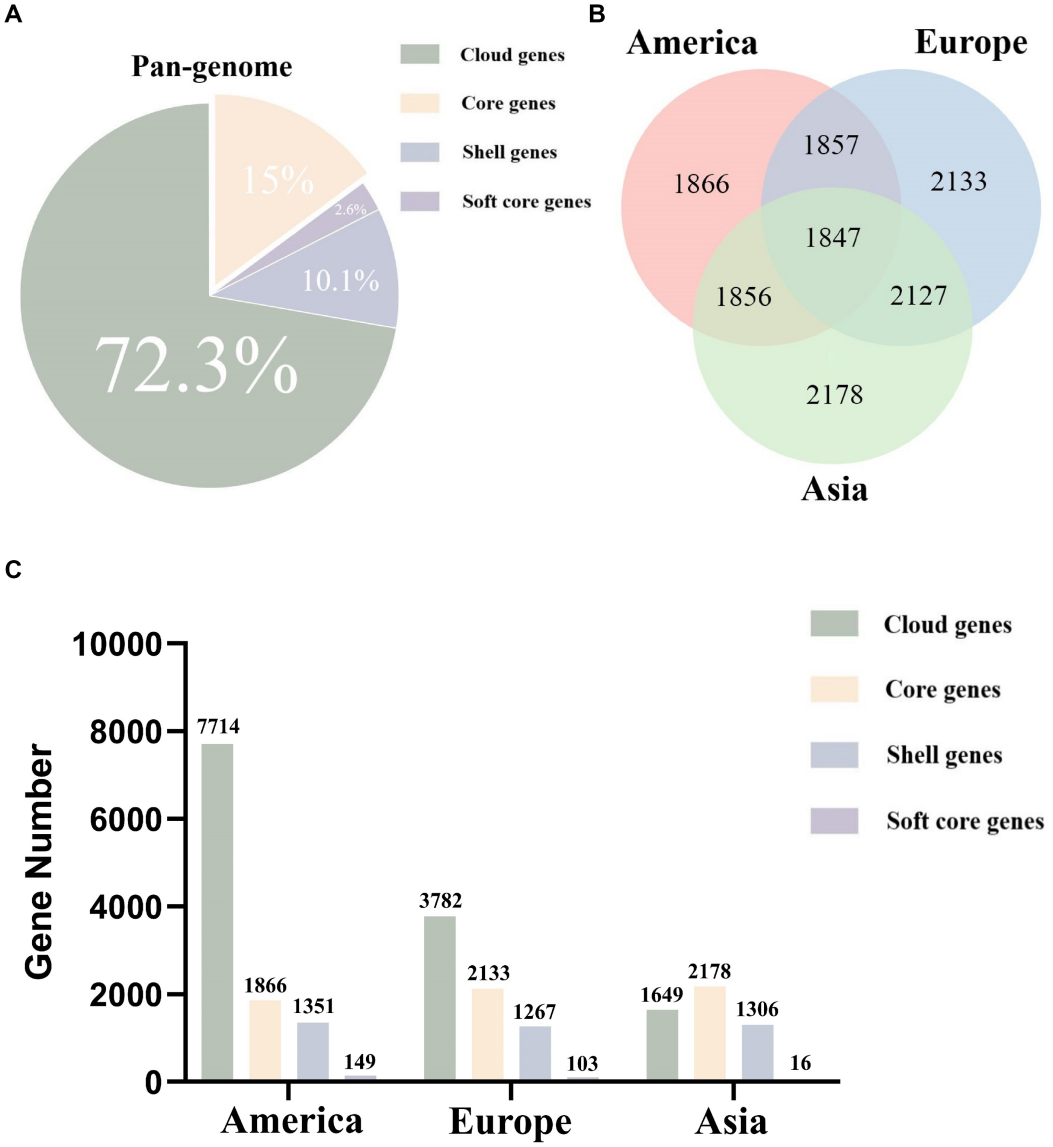
The proportion and quantity of various parts of the pan-genome in *L. monocytogenes* from different regions as analyzed through pan-genomics. **(A)** The pan-genome proportion of *L. monocytogenes* in three different regions. **(B)** The core genome Venn diagram of *L. monocytogenes* in three different regions. **(C)** The number of core genes, soft core genes, shell genes, and cloud genes in *L. monocytogenes* from three different regions.

Based on pan-genomic classification, the analysis of *L. monocytogenes* strains from different regions revealed the following gene distribution within the pan-genome: there were 1847 (15%) core genes, 314 (2.6%) soft-core genes, 1,237 (10.1%) shell genes, and 8,860 (72.3%) cloud genes (Figure 1A). The pan-genomic composition of *L. monocytogenes* varies across different regions. In America, *L. monocytogenes* strains possess 1866 core genes, 149 soft-core genes, 1,351 shell genes, and 7,714 cloud genes. In Europe, *L. monocytogenes* strains possess 2,133 core genes, 103 soft-core genes, 1,267 shell genes, and 3,782 cloud genes. In Asia, *L. monocytogenes* strains possess 2,178 core genes, 16 soft-core genes, 1,306 shell genes, and 1,649 cloud genes (Figures 1B,C). By analyzing and summarizing the genomic, pan-genomic, and core genomic features of *L. monocytogenes* strains from different regions, it was revealed that these strains possess open genomes, which provide a genetic basis for their adaptation to diverse environments.

### Pan-genomic analysis of *Listeria monocytogenes* strains from different regions and non-target bacterial strains for the screening of potential target genes

To identify potential target genes in *L. monocytogenes* strains from different regions, we conducted pan-genomic analysis of these strains as well as non-target bacterial strains. Among them, due to the presence of non-target strains, the quantities of core genes are 0, the quantity of soft-core genes are 1919, the quantity of shell genes are 1,458, the quantity of cloud genes are 41,390, and the total quantity of genes are 44,767. A total of 357 potential target genes were detected in *L. monocytogenes* strains from different regions (Supplementary Table S3). These potential target genes were present in the *L. monocytogenes* strains included in this study, while being absent in non-target bacterial strains investigated. These potential target genes have the potential to serve as novel target genes for *L. monocytogenes* strains in different regions, but further screening of these potential target genes is still required.

### MLST and phylogenetic analysis

**Figure 2:**
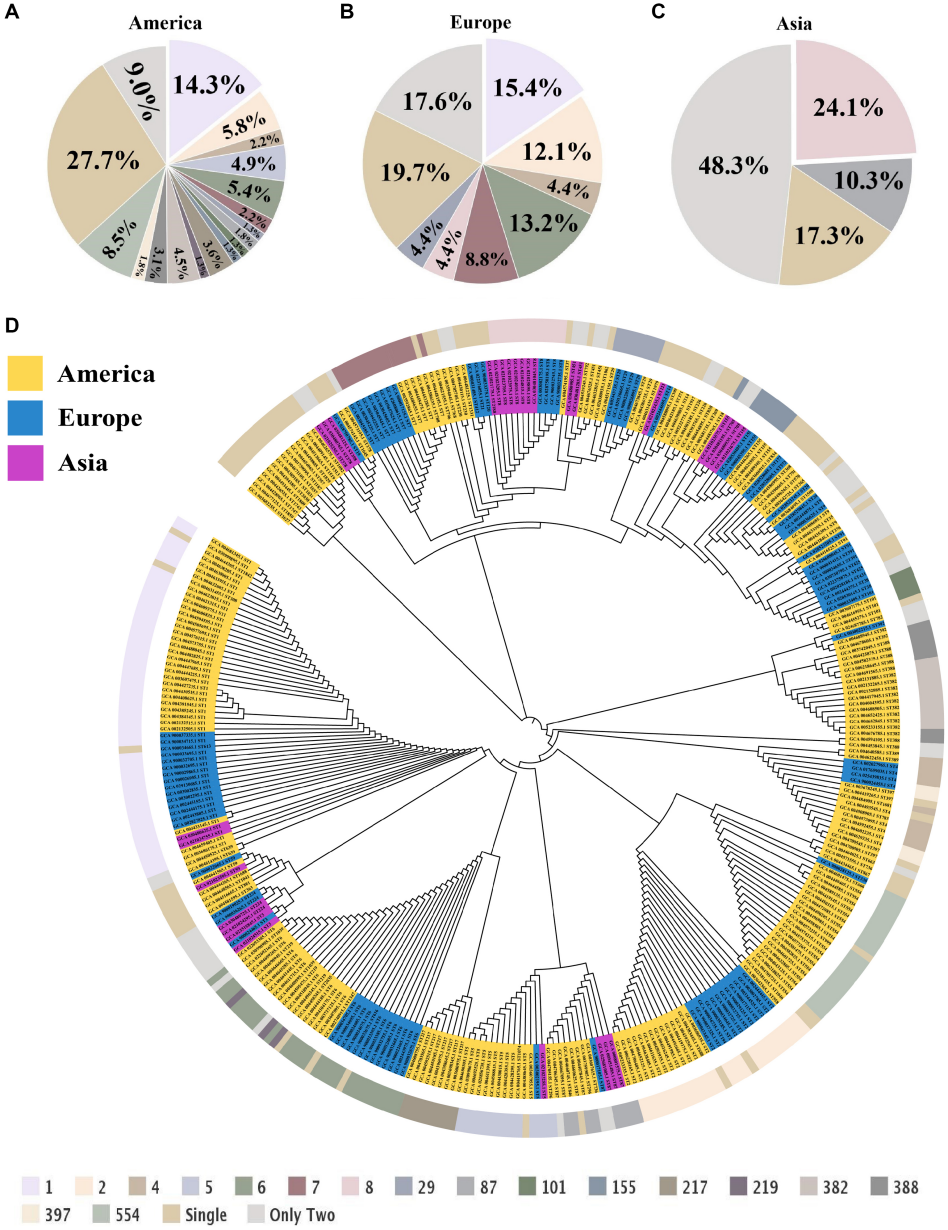
Multilocus sequence typing and phylogenetic tree of *L. monocytogenes* from different regions. **(A)** The proportion of STs in *L. monocytogenes* from the Americas region. **(B)** The proportion of STs in *L. monocytogenes* from the European region. **(C)** The proportion of STs in *L. monocytogenes* from the Asian region. **(D)** Phylogenetic trees of *L. monocytogenes* from different regions, with the outer circle indicating the corresponding ST for each strain.

To investigate the correlation among *L. monocytogenes* strains from different regions, MLST was employed to genotype the strains at the whole-genome level, aiming to determine the phylogenetic relationships among different sequence types (STs) and their associations with the disease. In America, the most common among *L. monocytogenes* strains was ST1 (*n* = 32, 14.3%), Clonal Complex 1 (CC1) (*n* = 34, 15.2%) and Lineage I (*n* = 161, 72.2%) (Figure 2A; Supplementary Table S4). In Europe, the most common was ST1 (*n* = 14, 15.4%), CC1 (*n* = 14, 15.4%) and Lineage I (*n* = 52, 57.1%) (Figure 2B; Supplementary Table S4). In Asia, the most common was ST8 (*n* = 7, 24.1%), CC8 (*n* = 8, 27.6%) and Lineage II (*n* = 18, 62.1%) (Figure 2C; Supplementary Table S4). In strains of *L. monocytogenes* in America and Europe, a higher proportion is observed for strains of ST1 and CC1 types. In strains of *L. monocytogenes* in Asia, a higher proportion is observed for strains of ST8 and CC8 types.

Medically intriguingly, *Listeria* species inhabit diverse ecological niches, but only *L. monocytogenes* and *L. ivanovii* exhibit pathogenicity (Orsi and Wiedmann, 2016; Lu et al., 2022). To elucidate the evolutionary patterns of *L. monocytogenes* strains from different regions, we conducted a phylogenetic analysis of *L. monocytogenes* using conserved amino acid sequences of all single-copy genes (Figure 2D). Through the phylogenetic tree analysis, we can observe that *L. monocytogenes* strains in different regions share a common ancestor, all falling within this major root of the *Listeria* genus. Although the geographical locations of the *L. monocytogenes* strains we studied vary significantly, some strains from different locations still cluster within the same branch, indicating they share a relatively similar phylogenetic relationship. This indicates that despite being in different geographic locations and under varying environmental conditions, *L. monocytogenes* exhibits certain similarities in evolutionary mechanisms and genetic variations in response to environmental pressures, displaying a strong adaptability to diverse environments.

### Enrichment analysis of the functional characteristics of potential target genes using GO and KEGG

**Figure 3:**
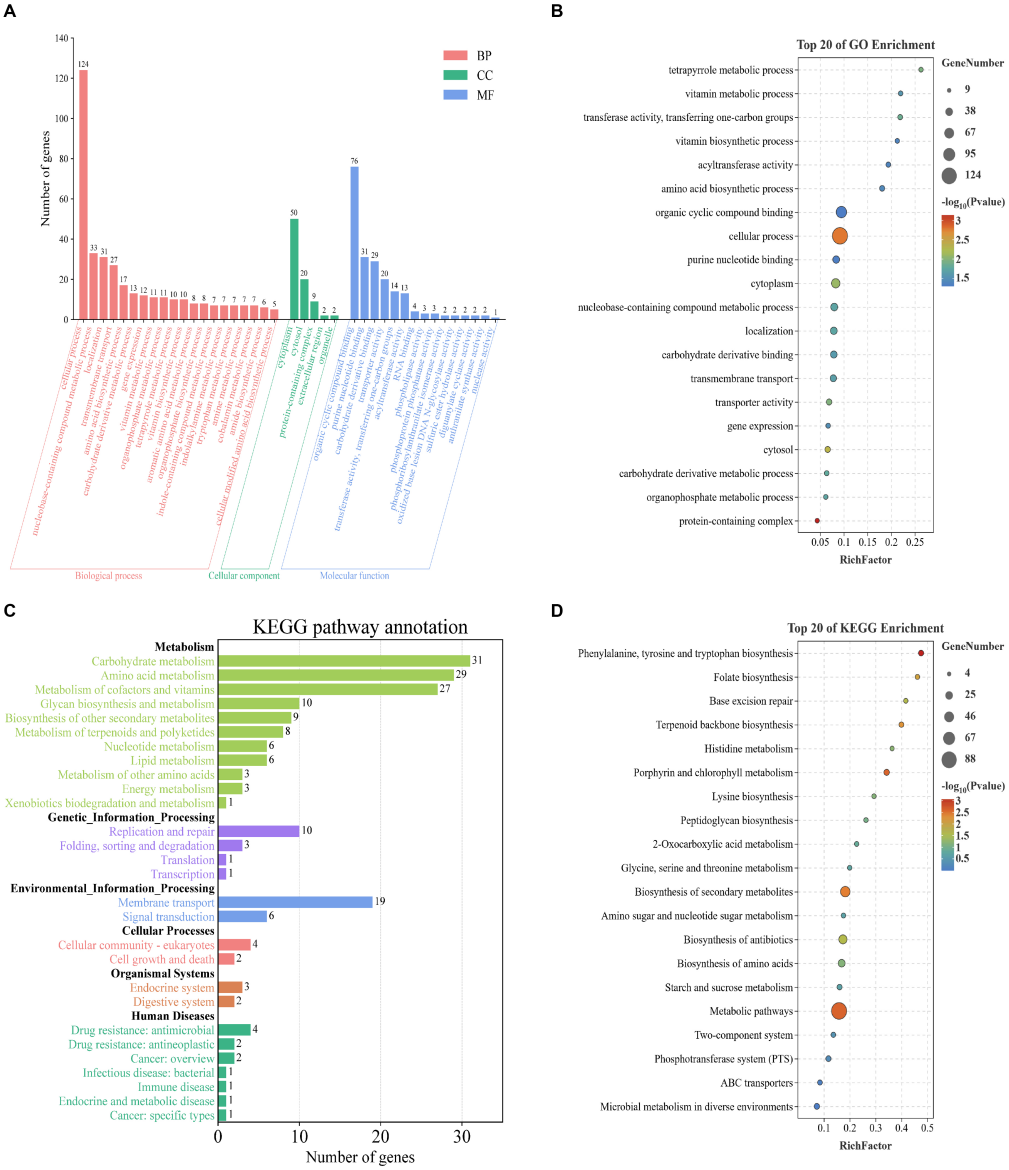
Enrichment analysis of potential target genes in *L. monocytogenes* from different regions based on the GO, and KEGG databases. **(A)** Enrichment analysis based on the GO database. **(B)** Enrichment analysis based on GO database with the top 20 enriched terms listed. **(C)** Enrichment analysis based on the KEGG database. **(D)** Enrichment analysis based on KEGG database with the top 20 enriched terms listed.

To investigate the functional characteristics of 357 potential target genes in *L. monocytogenes* strains from different regions, we performed functional annotation and classification of these genes using GO and KEGG databases. The detailed information of the potential target genes is presented in Supplementary Table S3. The GO database categorizes gene functions into three main categories, namely Biological Processes (BP), Cellular Components (CC), and Molecular Functions (MF). In the BP category, the most enriched biological processes were cellular process (*n* = 124, 34.7%), nucleobase-containing compound metabolic process (*n* = 33, 9.2%), localization (*n* = 31, 8.7%), and transmembrane transport (*n* = 27, 7.6%). Within the CC category, the most abundant cellular components were cytoplasm (*n* = 50, 14%). In the MF category, the most enriched molecular functions were organic cyclic compound binding (*n* = 76, 21.3%), and purine nucleotide binding (*n* = 31, 8.7%) (Figure 3A). We integrated and ranked all the GO enrichment analysis results of potential target genes, and generated a bubble chart (Figure 3B) to display the top 20 functional features in GO enrichment analysis based on the number of genes and the significance of *p* values.

The pathway database of KEGG is the most widely used public database for metabolic pathways, which classifies biological metabolic pathways into six categories: Metabolism, Genetic Information Processing, Environmental Information Processing, Cellular Processes, Organismal Systems, and Human Diseases. The potential target genes were annotated using KEGG in six categories. Among these, the most enriched pathways in the Metabolism category were carbohydrate metabolism (*n* = 31, 8.7%), amino acid metabolism (*n* = 29, 8.1%), and metabolism of cofactors and vitamins (*n* = 27, 7.6%). In the Genetic Information Processing category, replication and repair (*n* = 10, 2.8%) were the most enriched pathways. In the Environmental Information Processing category, membrane transport (*n* = 19, 5.3%) and signal transduction (*n* = 6, 1.7%) were the most enriched pathways. In the Cellular Processes category, cellular community – prokaryotes (*n* = 4, 1.1%) and cell growth and death (*n* = 2, 0.6%) were the most enriched pathways. In the Organismal Systems category, endocrine system (*n* = 3, 0.8%) and digestive system (*n* = 2, 0.6%) were the most enriched pathways. In the Human Diseases category, drug resistance: antimicrobial (*n* = 4, 1.1%) and drug resistance: antineoplastic (*n* = 2, 0.6%) were the most enriched pathways (Figure 3C). We integrated and ranked all the KEGG enrichment analysis results of potential target genes, and generated a bubble chart (Figure 3D) to display the top 20 functional features in KEGG enrichment analysis based on the number of genes and the significance of *p* values.

In summary, the enrichment analysis of functional characteristics of 357 potential target genes using GO and KEGG databases indicates that these genes are primarily associated with metabolic processes, compound binding, protein localization, and transmembrane transport in *L. monocytogenes*. Examples include cellular metabolic process, carbohydrate metabolism and organic substance biosynthetic process. However, there were still some genes with unclear functional information, which warrants further investigation in future studies. The potential target genes are closely associated with fundamental biological processes and infection pathogenesis of *L. monocytogenes*, playing crucial roles in sustaining basic life activities, invading the host, and exerting pathogenic effects.

### PPI network analysis of potential target genes and identification of novel target genes

The PPI network plays a crucial role in various biological processes within organisms. To further assess the interconnections among potential target genes of *L. monocytogenes* strains in different regions, PPI analysis was carried out using the STRING database. The PPI network of potential target genes comprised 357 genes, and visualization of the PPI network was performed using Cytoscap_v3.10.1 software. They were clustered together, indicating strong physical interaction or functional association.

**Figure 4:**
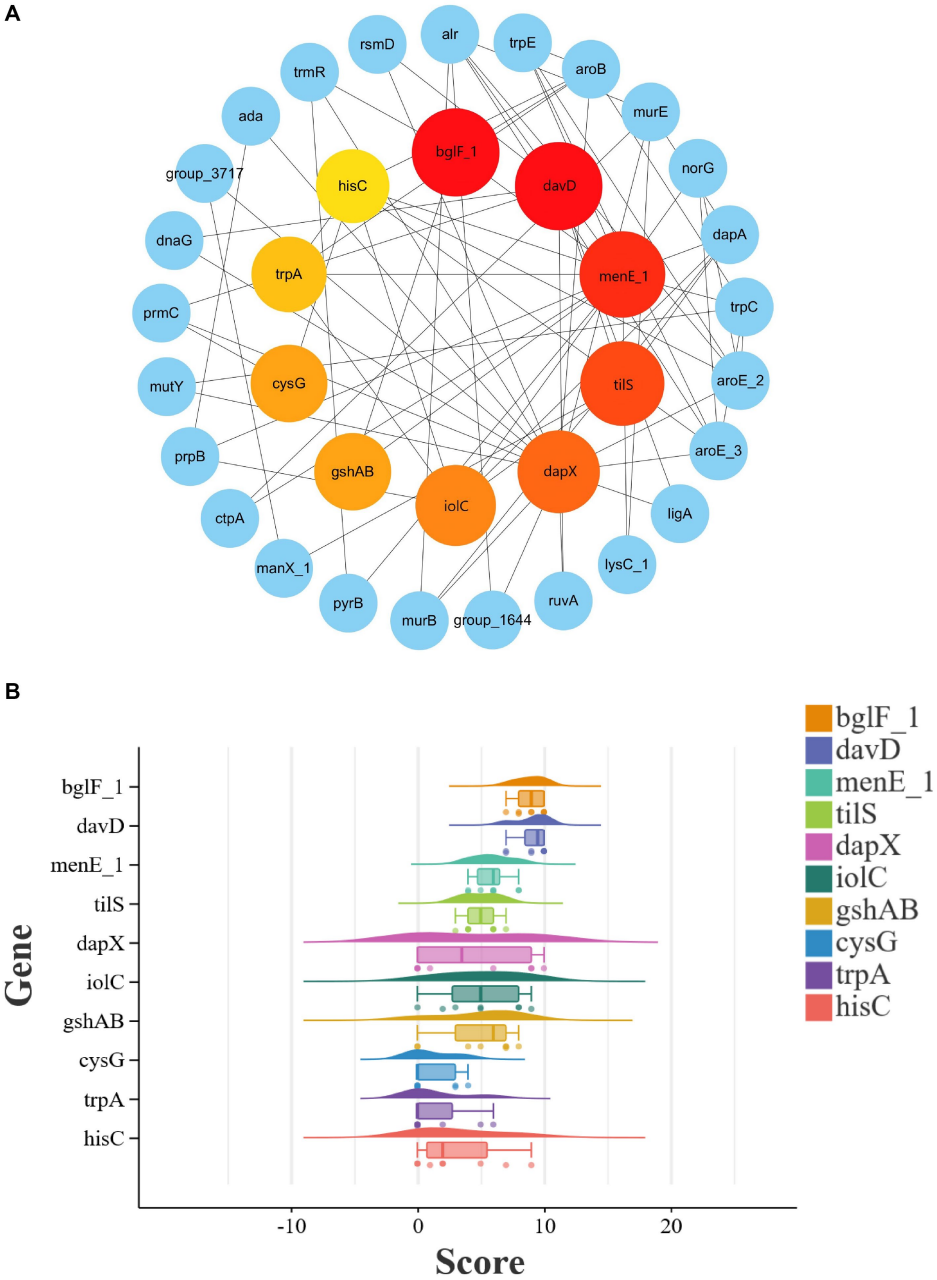
PPI network analysis and identification of novel target genes among potential target genes in *L. monocytogenes* from different regions. **(A)** Visualization of PPI network among the top 10 hub genes ranked by Degree algorithm. The larger the circle and the deeper the red color, the higher the score of the gene. **(B)** Scoring of hub genes across eight different algorithms.

To further analyze potential target genes of *L. monocytogenes* for the selection of novel target genes, we employed the CytoHubba function of Cytoscape v3.10.1 software to identify hub genes. Hub genes are key factors in protein-protein interaction networks that exhibit high connectivity in gene expression networks, indicating their ability to regulate multiple genes. The genes in the PPI network were screened using eight different algorithms available in the CytoHubba function. The top 10 genes with the highest scores were selected as hub genes, and their ranking is presented in Table 2. A comprehensive analysis of the results obtained from the eight algorithms was performed, and raincloud plots illustrating the scores of the hub genes for each algorithm was generated (Figure 4B). The top 10 genes with the highest scores identified by the Degree algorithm were ultimately determined as novel target genes, and a PPI network was constructed based on their scores (Figure 4A). Among them, *bglF_1* and *davD* genes had the highest score of 54, followed by *menE_1* gene with a score of 52, *tilS* gene with a score of 50, *dapX* gene with a score of 48, *iolC* gene with a score of 46, *gshAB* gene with a score of 42, *cysG* gene with a score of 42, *trpA* gene with a score of 40, and *hisC* gene with a score of 38. The detailed information regarding these 10 genes, including their functional roles, gene lengths, etc., is provided in Table 3. These 10 hub genes play crucial roles in sustaining basic life activities and infection invasion of *L. monocytogenes* strains, with the potential to become novel target genes for *L. monocytogenes* strains, particularly the top-scoring genes, *bglF_1* and *davD*.

**Table 2:** Top 10 hub genes ranked by scoring in eight different algorithms.

Catelogy	Rank methods in cytoHubba							
	Degree	Betweenness	BottleNeck	Closeness	EPC	MNC	Radiality	Stress
Gene symbol top 10	bglF_1^a^	bglF_1^a^	davD^b^	davD^b^	dapX	davD^b^	bglF_1^a^	davD^b^
	davD^b^	davD^b^	bglF_1^a^	dapX	hisC	iolC	dapX	bglF_1^a^
	menE_1	iolC	group_1214	bglF_1^a^	bglF_1^a^	menE_1	gshAB	iolC
	tilS	gshAB	gshAB	gshAB	davD^b^	bglF_1^a^	davD^b^	hisC
	dapX	tilS	menE_1	tilS	menE_1	trpA	tilS	menE_1
	iolC	menE_1	hisC	iolC	trpA	hisC	group_1214	gshAB
	gshAB	fni	tilS	menE_1	tilS	tilS	menE_1	cysG
	cysG	cysG	iolC	fni	trpF	ezrA	fni	tilS
	trpA	group_32878	group_8648	hisC	iolC	divIB	hisC	yodC
	hisC	dapX	yceM	group_1214	norG	trpF	hepS	group_3718

^a, b^The two genes highlighted in bold are the most promising candidates to serve as novel target genes.

**Table 3:** Detailed information of top 10 hub genes ranked by scoring in Degree algorithm.

Gene	Name of target genes	Sequence length/bp	Presence profile		Encoded protein	Product size/bp	Source
			In target	In non-target			
bglF_1	lmo1035	1857	343 (100%)	0	PTS system beta-glucoside-specific EIIBCA component	618	This study
davD	lmo0913	1,467	343 (100%)	0	Glutarate-semialdehyde dehydrogenase	488	This study
menE_1	lmo1672	1,409	343 (100%)	0	O-succinylbenzoic acid--CoA ligase	469	This study
tilS	lmo0219	1946	343 (100%)	0	tRNA(Ile)-lysidine synthase	648	This study
dapX	lmo1006	1,146	343 (100%)	0	Putative N-acetyl-LL-diaminopimelate aminotransferase	381	This study
iolC	lmo0385	978	343 (100%)	0	5-dehydro-2-deoxygluconokinase	325	This study
gshAB	lmo2770	2,331	343 (100%)	0	Glutathione biosynthesis bifunctional protein GshAB	776	This study
cysG	lmo1201	1,481	343 (100%)	0	Siroheme synthase	493	This study
trpA	lmo1627	774	343 (100%)	0	Tryptophan synthase alpha chain	257	This study
hisC	lmo1925	1,083	343 (100%)	0	Histidinol-phosphate aminotransferase	360	This study

### Distribution of virulence genes and antibiotic resistance genes in *Listeria monocytogenes* strains in different regions

To investigate the relationship between *L. monocytogenes* strains in different regions and their pathogenic mechanisms, we predicted virulence factor-encoding genes of the entire genome of *L. monocytogenes*. Based on VFDB prediction and annotation, virulence factors of *L. monocytogenes* were classified into 12 categories including Adherence, Bile resistance, Enzyme, Immune modulator, Intracellular survival, Invasion, Iron uptake, Nucleation-promoting factor, Peptidoglycan modification, Regulation, Surface protein anchoring, and Toxin.

**Figure 5:**
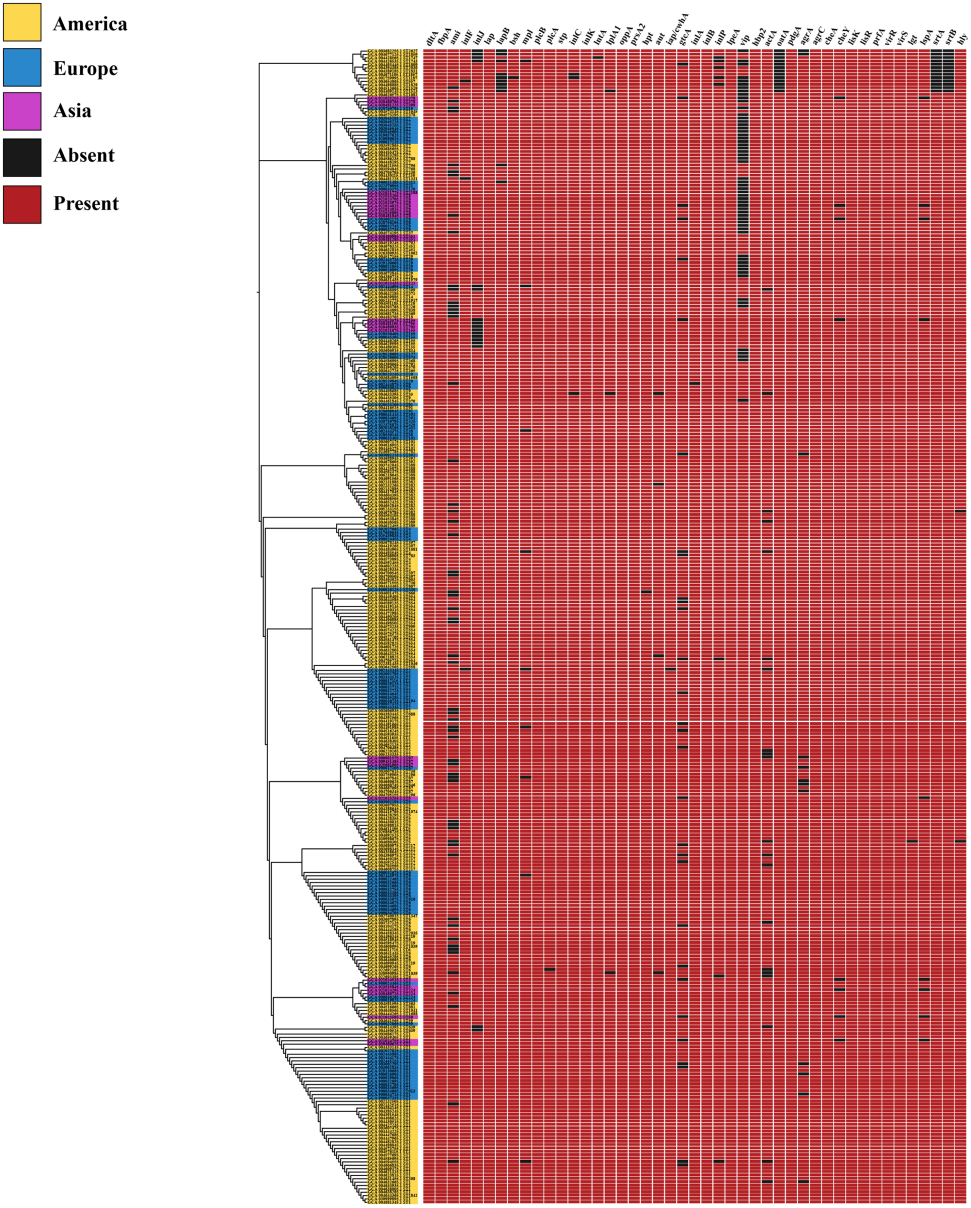
The distribution of virulence genes in *L. monocytogenes* from different regions.

In this study, the virulence genes *dltA*, *fbpA*, *lap*, *plcB*, *stp*, *inlK*, *oppA*, *prsA2*, *inlB*, *lpeA*, *hbp2*, *pdgA*, *agrC*, *cheA*, *lisK*, *lisR*, *prfA*, *virR*, and *virS* were found to be present in 100% of *L. monocytogenes* strains from different regions (Figure 5). Our predictive findings indicate the presence of numerous virulence genes, including *inlB*, and *plcB*, which play pivotal roles in *L. monocytogenes* infection and host invasion, across *L. monocytogenes* strains from various regions. It is well known that *L. monocytogenes* is pathogenic within the *Listeria* genus, owing to its abundance of virulence genes, which contribute to its pathogenicity. Through the prediction of virulence genes, *L. monocytogenes* strains from different regions exhibit high pathogenicity.

**Figure 6:**
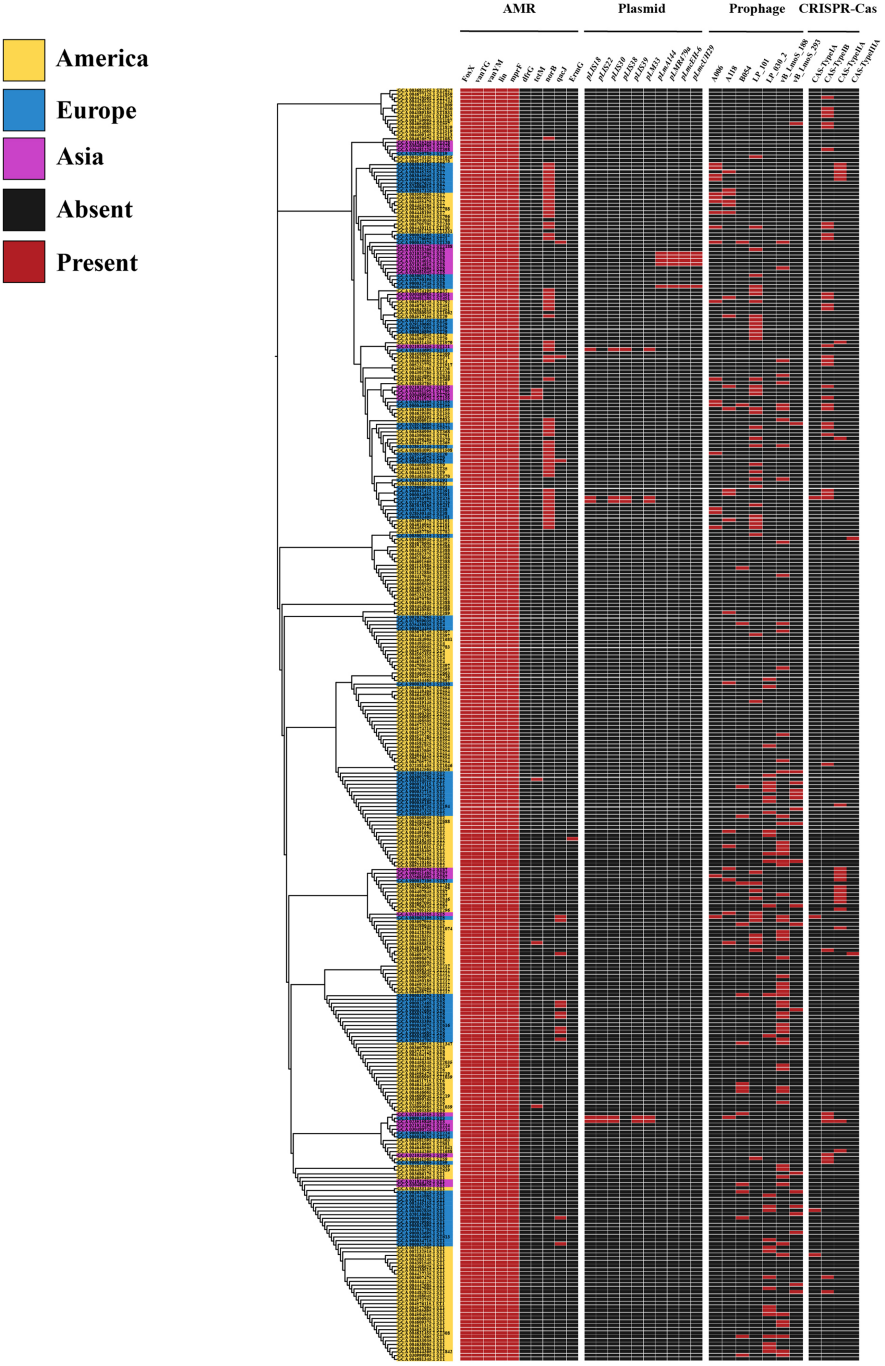
The distribution of antibiotic resistance genes, plasmids, prophages, and CRISPR-Cas systems in *L. monocytogenes* from different regions.

With the widespread use of antibiotics, the antimicrobial resistance of foodborne strains has increased in many countries. The distribution of antimicrobial resistance genes in *L. monocytogenes* was investigated using the CARD database. In this study, a total of 10 antimicrobial resistance genes belonging to 9 drug classes and exhibiting 5 resistance mechanisms were identified among the 343 *L. monocytogenes* genomes analyzed. Among the *L. monocytogenes* strains from different regions, 100% were found to harbor four types of antibiotic resistance genes, including phosphonic acid antibiotic gene (*FosX*), glycopeptide antibiotic genes (*vanTG*, *vanYM*), lincosamide antibiotic gene (*lin*), and peptide antibiotic gene (*mprF*) (Figure 6).

### Distribution of MGEs in *Listeria monocytogenes* strains in different regions

Currently, many scientists have begun to pay extensive attention to the significant role of horizontal transfer of MGEs in bacterial genome evolution and adaptation to specific environmental pressures. We employed PLSDB and PHASTER databases for the detection of MGEs, and the results only documented plasmids with an identity score of 1 and intact prophage regions. In this study, the *L. monocytogenes* genome contained a total of 10 plasmids and 7 intact prophage regions. Among them, plasmids *pLmA144*, *pLMR479a*, *pLmcEH-6*, and *pLmcUH29* were exclusively present in ST8, while plasmids *pLIS22* and *pLIS39* were only found in ST3 (Figure 6). The most prevalent prophage was PHAGE_Lister_vB_LmoS_188 [NC_028871] (*n* = 64, 18.7%), followed by PHAGE_Lister_LP_101 [NC_024387] (*n* = 48, 14%) (Figure 6). Intriguingly, despite the geographical disparity of *L. monocytogenes* isolates, identical phage genomes have been detected, suggesting a certain degree of similarity in MGEs across strains from different regions.

### Distribution of CRISPR-Cas system types in *Listeria monocytogenes* strains in different regions

The CRISPR-Cas system is a bacterial adaptive immune system that protects bacteria from viral infections, which is also associated with the virulence and pathogenicity of pathogens. In this study, we characterized the CRISPR-Cas systems in 343 *L. monocytogenes* genomes and identified four types of CRISPR-Cas systems. Each CRISPR-Cas system type exhibited distinct cas genes, with a total of 14 cas genes detected (Table 4). The CRISPR-Cas system types detected in *L. monocytogenes* included CAS-TypeIA (4/343), CAS-TypeIB (39/343), CAS-TypeIIA (21/343), and CAS-TypeIIIA (2/343). Approximately one-fifth of the *L. monocytogenes* genomes (66/343) harbored at least one CRISPR-Cas system, with CAS-TypeIB (11.4%) and CAS-TypeIIA (6%) being the most prevalent (Figure 6). CAS-TypeIA was only detected in ST1, ST5, and ST425, while CAS-TypeIIIA was found exclusively in ST5 and ST392. The CAS-type IA system detected in the *L. monocytogenes* isolates in this study was composed of csa5_TypeIA and casRa_TypeIA. The CAS-TypeIB system was composed of cas5b_TypeIB, cas6_TypeI-III, cas8a1b_TypeIB, cas7b_TypeIB, cas3_TypeI, cas2_TypeI-II-III, cas4_TypeI-II, and cas1_TypeIB. The CAS-TypeIIA system was composed of csn2_TypeIIA, cas2_TypeI-II-III, cas1_TypeII, and cas9_TypeII. The CAS-type IIIA system was composed of csm2_TypeIIIA.

**Table 4:** Identification of CRISPR-Cas system types and the corresponding cas genes detected in *L. monocytogenes* strains from different regions.

Types	Cas genes	Area	Number	Total
CAS-type IA	csa5_TypeIA	America	1	2
		Europe	1	
		Asia	0	
CAS-type IA	casRa_TypeIA	America	0	2
		Europe	2	
		Asia	0	
CAS-TypeIB	cas5b_TypeIB	America	3	5
		Europe	1	
		Asia	1	
CAS-TypeIB	cas6_TypeI-III, cas8a1b_TypeIB, cas7b_TypeIB, cas5b_TypeIB, cas3_TypeI, cas2_TypeI-II-III	America	8	13
		Europe	3	
		Asia	2	
CAS-TypeIB	cas6_TypeI-III, cas8a1b_TypeIB, cas7b_TypeIB, cas5b_TypeIB, cas3_TypeI, cas4_TypeI-II, cas1_TypeIB, cas2_TypeI-II-III	America	9	21
		Europe	6	
		Asia	6	
CAS-type IIA	csn2_TypeIIA, cas2_TypeI-II-III, cas1_TypeII, cas9_TypeII	America	11	22
		Europe	7	
		Asia	4	
CAS-type IIIA	csm2_TypeIIIA	America	2	2
		Europe	0	
		Asia	0	
Total				67

### Detection of *Listeria monocytogenes* using specific primers by PCR

**Figure 7:**
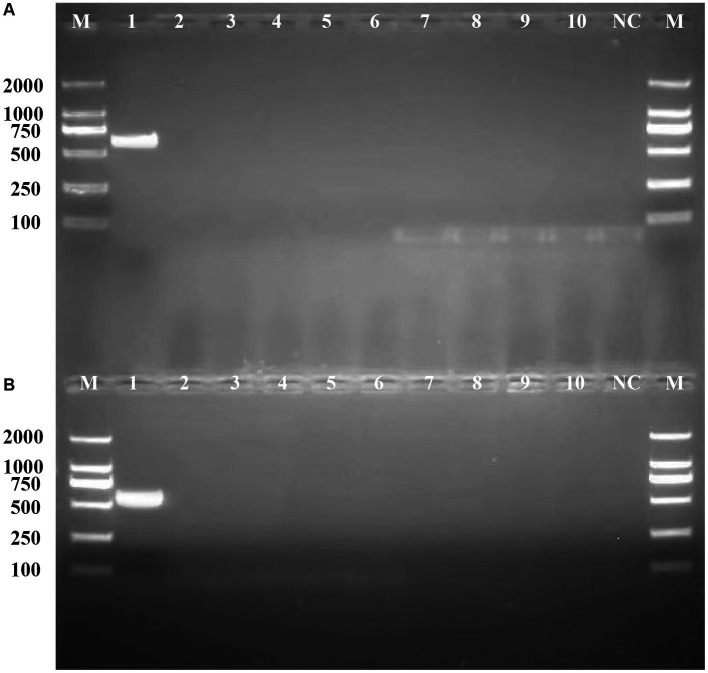
Validation of the specificity of the primers for the *bglF_1* and *davD* genes in *L. monocytogenes*. **(A)** The PCR results of the primer system targeting the *bglF_1* gene. **(B)** The PCR results of the primer system targeting the *davD* gene. Lane M: DL DNA 2000 marker, lane NC: negative control, and lanes 1–10: represent 10 different strains, including lane 1: *Listeria monocytogenes*, lane 2: *Listeria innocua*, lane 3: *Listeria ivanovii*, lane 4: *Listeria welshimeri*, lane 5: *Escherichia coli*, lane 6: *Salmonella*, lane 7: *Klebsiella Pneumoniae*, lane 8: *Acinetobacter baumannii*, lane 9: *Pseudomonas aeruginosa*, lane 10: *Pneumocystosis jirovecii*.

To validate the potential of *bglF_1* and *davD* genes as specific molecular detection and therapeutic targets in *L. monocytogenes* strains from different regions, primers were designed for *bglF_1* and *davD* genes, followed by PCR experiments to assess their specificity. The PCR results revealed a distinct band at 616 bp for *L. monocytogenes* in the *bglF_1* gene primer system, while *non-L. monocytogenes* samples showed no band (Figure 7A). Similarly, a clear band at 567 bp was observed for *L. monocytogenes* in the *davD* gene primer system, with no band detected in *non-L. monocytogenes* samples (Figure 7B). The results demonstrated the excellent specificity of the *bglF_1* and *davD* genes for *L. monocytogenes*. Therefore, the *bglF_1* and *davD* genes hold promise as specific molecular detection and therapeutic targets for *L. monocytogenes* strains from different regions.

## Discussion

*L. monocytogenes*, as a significant foodborne pathogen, is widely prevalent worldwide, posing a serious threat to human life and health. Therefore, we conducted comparative genomic analysis of *L. monocytogenes* strains from different regions to explore their biodiversity and evolutionary characteristics, identify potential target genes, and further mining novel target genes, aiming to provide novel specific molecular detection and therapeutic strategies for *L. monocytogenes* strains.

In this study, we conducted a pan-genomic comparative analysis of 343 *L. monocytogenes* strains from different regions to investigate the biodiversity and evolutionary characteristics of strains. To assess the genomic biodiversity of *L. monocytogenes* strains in different regions, we conducted core/pan-genome analysis. Core and accessory genomes were analyzed based on the whole genomes of *L. monocytogenes*. The core genome represents the essential portion necessary for the presence and shared phenotypic features of specific strains, while the accessory genome provides unique characteristics for a species or strain that are not essential for their basic survival, but offer selective advantages for ecological adaptation and antibiotic resistance (Deng et al., 2010; Zhang et al., 2017; Lu et al., 2022). Although the studied strains of *L. monocytogenes* are geographically diverse, there still exist 1847 core genes that constitute the fundamental components of *L. monocytogenes* survival and development. In America, the number of core genes in *L. monocytogenes* strains is 1866, in Europe it is 2,133, and in Asia it is 2,178. It can be observed that the number of core genes in *L. monocytogenes* strains varies across different regions. Apart from the core genes they share, *L. monocytogenes* strains in different regions also possess unique core genes that are present only in one region and absent in others. These unique core genes may be the primary reason for the distinctiveness of *L. monocytogenes* strains in one region compared to those in other regions. The primary reason for this phenomenon may be attributed to the different environments in which the strains reside. Hence, in order to adapt to these unique environments, the strains have evolved genes that are specific to these environments to counteract environmental pressures (Liao et al., 2023). This is also very intriguing, as it allows for the exploration of the differences among *L. monocytogenes* strains in different regions, analyzing the unique characteristics of strains in different areas, thereby studying the evolutionary patterns of strains in that region, and subsequently devising targeted prevention and control measures for that region. As the number of genomes increases, the pan-genome size continues to rise while the core genome decreases and tends to plateau. This indicates that the studied *L. monocytogenes* possesses an open pan-genome, which provides a genetic basis for the adaptation of *L. monocytogenes* to different environments. The potential target genes are exclusively present in *L. monocytogenes* strains in different regions, while they are absent in non-target strains. This indicates that the potential target genes play a crucial role in the pathogenicity of *L. monocytogenes* strains in different regions. These genes are indispensable for the survival, virulence, and invasion of *L. monocytogenes*, making them essential for maintaining life activities and infection. Therefore, investigating potential target genes can facilitate the analysis of the biodiversity and evolutionary characteristics of *L. monocytogenes*, aiding in the selection of novel specific molecular detection and therapeutic target genes.

To investigate the biodiversity and evolutionary characteristics of *L. monocytogenes* strains in different regions, we conducted MLST typing analysis. The results revealed that *L. monocytogenes* strains from America and Europe were predominantly characterized by ST1 and CC1 types, whereas those from Asia were predominantly characterized by ST8 and CC8 types. Wang et al. (2012) identified the three most common *L. monocytogenes* types in China as ST8, ST9, and ST87. Amarasekara et al. (2024) identified a significant presence of ST1 and CC1 types among *L. monocytogenes* isolates from agricultural markets in the United States. Additionally, Toledo et al. (2018) found that the most prevalent type of *L. monocytogenes* in samples from Chile was ST1. Our results are consistent with the findings reported in the above-mentioned literature. Our findings indicate that the *L. monocytogenes* strains isolated from America, Europe, and Asia exhibit different types. The underlying reasons for this phenomenon could be attributed to variations in the transmission routes and environmental conditions of *L. monocytogenes*, as well as genetic variability among strains. Based on our analysis, although *L. monocytogenes* strains originate from diverse geographical regions, they exhibit relatively similar phylogenetic relationships in the constructed phylogenetic tree. This indicates that while the *L. monocytogenes* strains are present in different environments, they exhibit a certain degree of genetic similarity in terms of bacterial variability. Under various environmental pressures, *L. monocytogenes* gradually evolves into life forms adapted to these specific environments, undergoing extensive genetic variations. Bacterial genetic variations result in distinct predominant types of *L. monocytogenes* strains in America, Europe, and Asia. Interestingly, the predominant sequence type of *L. monocytogenes* in both America and Europe is ST1, which may be attributable to the relatively close geographical proximity of these regions resulting in fewer environmental disparities. Additionally, trade between these regions may contribute to the mutual dissemination of *L. monocytogenes* strains. This discovery provides valuable insights into the reasons for the differences in the predominant ST and CC types of *L. monocytogenes* strains in America, Europe, and Asia.

It is well known that within the genus *Listeria*, only *L. monocytogenes* and *L. ivanovii* are considered pathogenic, with *L. monocytogenes* exhibiting higher pathogenicity. Moreover, the high pathogenicity of *L. monocytogenes* typically relies on a plethora of virulence genes as its foundation. Our analysis findings align with this observation, as strains of *L. monocytogenes* in different regions harbor a significant abundance of virulence genes. During invasion of the host by *L. monocytogenes*, the bacterium first utilizes the *inlA* and *inlB* genes to bind with the E-Cadherin and Met receptors of the host’s eukaryotic cell membrane, respectively, thereby inducing bacterial uptake through receptor-mediated endocytosis. After internalization, the bacterium is encapsulated within a vacuole, and releases the *hly*, *plcA*, and *plcB* genes to mediate vacuole escape. Subsequently, the *actA* gene is utilized to induce actin polymerization and generate sufficient force for the bacterium to spread from one cell to another. During the invasion process of *L. monocytogenes*, LIPI-1 (*prfA*, *plcA*, *hly*, *mpl*, *actA*, and *plcB*) and LIPI-2 (*inlA*, *inlB*, *inlC*, *inlE*, *inlF*, *inlG*, *inlH*, *inlJ*, and *inlK*) play pivotal roles (Mejía et al., 2023). Interestingly, in this study, the genes *prfA*, *plcB*, *inlK*, and *inlB* were found to be present in 100% of the selected *L. monocytogenes* strains, whereas *hly*, *actA*, and *inlA* were not always present at 100%, but their presence probability exceeded 99%. This phenomenon could be attributed to prediction errors in the database or possibly due to genetic variations occurring in individual strains under specific environmental conditions (Li et al., 2021d). These virulence genes are essential for infecting and invading hosts, highlighting the high pathogenicity of *L. monocytogenes*. Furthermore, the potential target genes we screened also include these virulence genes. The potential target genes play crucial roles in the fundamental life activities and infective invasion of *L. monocytogenes*. Selecting these virulence genes as molecular detection and therapeutic targets may be a viable option, however, it may lack novelty, as previous studies have validated genes such as *inlA*, *inlB*, and *hly* as targets for the detection and treatment of *L. monocytogenes*. Therefore, although these virulence genes were also selected as potential target genes in this study, they were not directly chosen as targets for detection and treatment. This study employed hub gene screening methods to further select hub genes from numerous potential target genes. As is well known, hub genes, also known as key genes, refer to genes that play a crucial role in a particular disease or biological process (Li et al., 2022). Therefore, we selected highly scoring hub genes from potential target genes as novel target genes.

In this study, *L. monocytogenes* strains demonstrated relatively high resistance to phosphonic antibiotics, glycopeptide antibiotics, lincosamide antibiotics, and peptide antibiotics. Therefore, it is recommended to avoid selecting these four classes of antibiotics when undergoing treatment. Other types of antibiotics may yield better therapeutic effects, such as ampicillin, gentamicin, and penicillin. Analyzing MGEs can provide insights into the evolution of bacterial genomes. In this study, despite the different geographical locations of the *L. monocytogenes* strains, they exhibited certain similarities at the MGEs level. This suggests that although *L. monocytogenes* is exposed to diverse external environments, there still exists a degree of similarity in terms of bacterial genome evolution.

By performing PPI network analysis and conducting GO and KEGG enrichment analyses on potential target genes, we aimed to understand the role of these genes in *L. monocytogenes* strains in different regions (Adnan et al., 2022). Functional annotation results revealed that the potential target genes encompassed a significant number of transport and metabolism genes, as well as virulence-associated genes, which play crucial roles in the fundamental life activities and pathogenicity of *L. monocytogenes*. However, some genes still lack clear functional information, necessitating further investigation in future studies. Hub genes, which are the most crucial genes in PPI networks, were selected to mining novel target genes (Li et al., 2022). Ten highly connected hub genes (*bglF_1, davD, menE_1, tilS, dapX, iolC, gshAB, cysG, trpA, hisC*) were identified from the pool of potential target genes. These ten hub genes play crucial roles in the fundamental life activities and infective invasion of *L. monocytogenes*. Among them, *bglF_1* and *davD* genes scored the highest and showed closer connections with other proteins, indicating their potential to serve as specific molecular detection and therapeutic targets for *L. monocytogenes* strains. The inhibitors or antagonists of these genes hold promise as novel therapeutic agents. The PCR results demonstrated the excellent specificity of the *bglF_1* and *davD* genes for *L. monocytogenes*. Therefore, the *bglF_1* and *davD* genes hold promise as specific molecular detection and therapeutic targets for *L. monocytogenes* strains from different regions.

## Conclusion

In summary, we employed comparative genomic analysis to investigate the biodiversity and evolutionary characteristics of *L. monocytogenes* strains from different regions. Although *L. monocytogenes* strains originate from different regions, they exhibit a high degree of similarity in bacterial genome evolution, harboring numerous potential target genes that sustain the essential life activities and infection invasion of *L. monocytogenes*. Through further exploration of potential target genes and validation of PCR results, the *bglF_1* and *davD* genes emerged as promising candidates for specific molecular detection and therapeutic targets in *L. monocytogenes* strains. This study provides significant reference value for the specific molecular detection and therapeutic targets of *L. monocytogenes* strains.

## Data availability statement

The original contributions presented in the study are included in the article/Supplementary material, further inquiries can be directed to the corresponding author.

## Author contributions

BZ: Data curation, Project administration, Validation, Visualization, Writing – original draft, Writing – review & editing. HR: Supervision, Writing – review & editing. XW: Supervision, Writing – review & editing. CH: Validation, Writing – original draft. YJ: Validation, Writing – original draft. XH: Validation, Writing – original draft. RS: Validation, Writing – original draft. CL: Validation, Writing – original draft. YW: Validation, Writing – original draft. YL: Supervision, Writing – review & editing. SL: Supervision, Writing – review & editing. ZL: Supervision, Writing – review & editing. PH: Supervision, Writing – review & editing.
