# Supplementary material for: Comparative genomics analysis to explore the biodiversity and mining novel target genes of Listeria monocytogenes strains from different regions
Source: Front Microbiol. 2024 Jun 19;15:1424868. doi: 10.3389/fmicb.2024.1424868 (PMC11220162; doi:10.3389/fmicb.2024.1424868)
Supplement: Supplementary file 1 [file Data_Sheet_1.pdf]

Supplementary Material

Comparative genomics analysis to explore the biodiversity and mining novel target genes of *Listeria monocytogenes* strains from different regions

Bo Zhang[1†], Honglin Ren[1†], Xiaoxu Wang[2†], Cheng Han[1], Yuanyuan Jin[1], Xueyu Hu[1], Ruoran Shi[1], Chengwei Li[1], Yuzhu Wang[1], Yansong Li[1], Shiying Lu[1], Zengshan Liu[1] and Pan Hu[1*]

[1]State Key Laboratory for Diagnosis and Treatment of Severe Zoonotic Infectious Diseases, Key Laboratory for Zoonosis Research of the Ministry of Education, Institute of Zoonosis, and College of Veterinary Medicine, Jilin University, Changchun 130062, China

[2]Institute of Special Animal and Plant Sciences of Chinese Academy of Agricultural Sciences, Changchun 130112, Jilin, China

***Corresponding author:**

Pan Hu

E-mail: hupan84@163.com

Telephone: +86 15004318401

[†]These authors contributed equally to this work and share first authorship.

Table S1. Detailed information of *L. monocytogenes* strains from different regions, other *Listeria* strains, and *non-Listeria* strains.

Region	Bacterial species	GenBank	Genome size (Mbp)	GC content(%)	No. of contigs	N50(Kbp)	Date	Isolation Source
America	Listeria monocytogenes （n=223）	GCA_030990985.1 GCA_030998675.1 GCA_030999895.1 GCA_026380935.1 GCA_025856735.1<br>GCA_024687785.1 GCA_022693385.1 GCA_022692165.1 GCA_022381435.1 GCA_004358645.2<br>GCA_004472985.1 GCA_004464625.1 GCA_004408625.1 GCA_004461845.1 GCA_004458345.1<br>GCA_003759905.1 GCA_003742045.1 GCA_003737125.1 GCA_004394385.1 GCA_004392565.1<br>GCA_003607955.1 GCA_003607895.1 GCA_003607615.1 GCA_003607475.1 GCA_003607175.1<br>GCA_003606935.1 GCA_003606915.1 GCA_004455475.1 GCA_004454895.1 GCA_004453845.1<br>GCA_003698345.1 GCA_003686175.1 GCA_003685655.1 GCA_003684095.1 GCA_003680975.1<br>GCA_003682255.1 GCA_003681735.1 GCA_003678245.1 GCA_003642565.1 GCA_003642725.1<br>GCA_003594045.1 GCA_003592865.1 GCA_004706485.1 GCA_004706345.1 GCA_004705155.1<br>GCA_004705725.1 GCA_004705705.1 GCA_004703565.1 GCA_004700505.1 GCA_004700545.1<br>GCA_004633395.1 GCA_004633935.1 GCA_004632815.1 GCA_004632805.1 GCA_004632665.1<br>GCA_004631455.1 GCA_004631435.1 GCA_004629595.1 GCA_004629335.1 GCA_004626575.1<br>GCA_005233155.1 GCA_004623815.1 GCA_004622455.1 GCA_004621555.1 GCA_004621315.1<br>GCA_002740915.1 GCA_004616955.1 GCA_004614395.1 GCA_004611635.1 GCA_004611715.1<br>GCA_004611395.1 GCA_004609375.1 GCA_004608995.1 GCA_004608755.1 GCA_004608505.1<br>GCA_004606835.1 GCA_004604395.1 GCA_004602235.1 GCA_004601725.1 GCA_004601475.1<br>GCA_004595475.1 GCA_004594555.1 GCA_004699265.1 GCA_002132885.1 GCA_002132515.1<br>GCA_002132505.1 GCA_002132265.1 GCA_002131885.1 GCA_005232775.1 GCA_004691565.1<br>GCA_004692525.1 GCA_004690945.1 GCA_004688945.1 GCA_004681345.1 GCA_004680305.1<br>GCA_004594105.1 GCA_004593935.1 GCA_004592515.1 GCA_004592455.1 GCA_004588815.1<br>GCA_004589135.1 GCA_004588095.1 GCA_004586995.1 GCA_004427235.1 GCA_004586235.1<br>GCA_004585695.1 GCA_004582375.1 GCA_004582825.1 GCA_004581595.1 GCA_004581475.1<br>GCA_001759995.1 GCA_004577265.1 GCA_004577695.1 GCA_005233335.1 GCA_004576115.1<br>GCA_004575375.1 GCA_004574195.1 GCA_004574215.1 GCA_004574145.1 GCA_004573755.1<br>GCA_004571555.1 GCA_004573195.1 GCA_004573235.1 GCA_004573095.1 GCA_004678605.1<br>GCA_006220165.1 GCA_006218825.1 GCA_006218645.1 GCA_004676785.1 GCA_004677125.1<br>GCA_004676325.1 GCA_004673845.1 GCA_004671105.1 GCA_004667095.1 GCA_004659405.1<br>GCA_004660745.1 GCA_004660625.1 GCA_004654065.1 GCA_004652425.1 GCA_004652125.1<br>GCA_004652845.1 GCA_004648565.1 GCA_004645285.1 GCA_004646665.1 GCA_004643125.1<br>GCA_004644585.1 GCA_004644365.1 GCA_004641565.1 GCA_004642185.1 GCA_004641445.1<br>GCA_004640585.1 GCA_004638405.1 GCA_004638285.1 GCA_004638005.1 GCA_004501185.1<br>GCA_004417945.1 GCA_004389185.1 GCA_004498505.1 GCA_004498885.1 GCA_004496985.1<br>GCA_004496265.1 GCA_004496345.1 GCA_004639885.1 GCA_004416525.1 GCA_004493545.1<br>GCA_004493445.1 GCA_004388645.1 GCA_004491985.1 GCA_004491665.1 GCA_004490205.1<br>GCA_004489305.1 GCA_004415765.1 GCA_004488045.1 GCA_004396975.1 GCA_004484905.1<br>GCA_004482825.1 GCA_004483765.1 GCA_004481225.1 GCA_004393765.1 GCA_004450525.1<br>GCA_004450315.1 GCA_004450185.1 GCA_004448195.1 GCA_004447965.1 GCA_004447685.1<br>GCA_004446265.1 GCA_004409145.1 GCA_004444265.1 GCA_004444225.1 GCA_004444185.1<br>GCA_004384145.1 GCA_004408685.1 GCA_004435305.1 GCA_004433145.1 GCA_004430515.1<br>GCA_004428355.1 GCA_004383445.1 GCA_004430115.1 GCA_004428565.1 GCA_004430015.1<br>GCA_004426535.1 GCA_004391545.1 GCA_004425875.1 GCA_004516245.1 GCA_004517155.1<br>GCA_004513665.1 GCA_004419175.1 GCA_004419145.1 GCA_004386725.1 GCA_004455275.1<br>GCA_004453285.1 GCA_004385245.1 GCA_004449405.1 GCA_004508905.1 GCA_004390665.1<br>GCA_004519245.1 GCA_004428295.1 GCA_004434465.1 GCA_004407845.1 GCA_004419265.1<br>GCA_004104175.1 GCA_004518945.1 GCA_004516665.1	2.8-3.2	37.5-38.0	⩽214	25.7-1500.0	2016-2023	Cerebrospinal fluid
Europe	Listeria monocytogenes （n=91）	GCA_900034675.1 GCA_900035185.1 GCA_900024465.1 GCA_900037335.1 GCA_900034655.1<br>GCA_900022055.1 GCA_900037325.1 GCA_900032745.1 GCA_900019025.1 GCA_900033375.1<br>GCA_900033385.1 GCA_900036735.1 GCA_900034685.1 GCA_900032735.1 GCA_900028125.1<br>GCA_900032685.1 GCA_900026895.1 GCA_900034715.1 GCA_900017115.1 GCA_900032705.1<br>GCA_900041415.1 GCA_900022465.1 GCA_900032695.1 GCA_900029125.1 GCA_900034695.1<br>GCA_900032715.1 GCA_900033395.1 GCA_900033405.1 GCA_900033675.1 GCA_900032675.1<br>GCA_900034705.1 GCA_900033695.1 GCA_900022065.1 GCA_900032755.1 GCA_900017105.1<br>GCA_900032665.1 GCA_900034665.1 GCA_900026905.1 GCA_900045395.1 GCA_900032725.1<br>GCA_900029865.1 GCA_900034645.1 GCA_900031485.1 GCA_900024455.1 GCA_900036625.1<br>GCA_900017125.1 GCA_900043345.1 GCA_900036265.1 GCA_003045605.1 GCA_003045545.1<br>GCA_003045255.1 GCA_003045225.1 GCA_003045185.1 GCA_002445155.1 GCA_002444875.1<br>GCA_002444695.1 GCA_002444445.1 GCA_002444375.1 GCA_002444175.1 GCA_002443975.1<br>GCA_002443885.1 GCA_002443735.1 GCA_028750785.1 GCA_028750195.1 GCA_028608045.1<br>GCA_028529845.1 GCA_028530145.1 GCA_028530405.1 GCA_028523205.1 GCA_028528605.1<br>GCA_028523245.1 GCA_028528685.1 GCA_030730795.1 GCA_029130685.1 GCA_029130665.1<br>GCA_026080615.1 GCA_025439835.1 GCA_022375875.1 GCA_022376055.1 GCA_019657625.1<br>GCA_017659035.1 GCA_003002835.1 GCA_003002785.1 GCA_003002295.1 GCA_003002215.1<br>GCA_003002195.1 GCA_003002175.1 GCA_002028185.1 GCA_002027965.1 GCA_002027825.1<br>GCA_003344525.1	2.9-3.2	37.5-38.0	⩽42	224.8-2900.0	2016-2023	Cerebrospinal fluid
Asia	Listeria monocytogenes （n=29）	GCA_030481285.1 GCA_030481225.1 GCA_030481195.1 GCA_030481045.1 GCA_030480985.1<br>GCA_030480935.1 GCA_023515845.1 GCA_021025895.1 GCA_021025255.1 GCA_030480625.1<br>GCA_021024815.1 GCA_021021775.1 GCA_021021675.1 GCA_000801575.1 GCA_021024755.1<br>GCA_021023705.1 GCA_021023435.1 GCA_021023255.1 GCA_021023205.1 GCA_021024015.1<br>GCA_021023975.1 GCA_021023595.1 GCA_021023575.1 GCA_021024515.1 GCA_021024295.1<br>GCA_030480765.1 GCA_030480725.1	2.8-3.1	38.0	⩽72	287.0-3000.0	2014-2023	Cerebrospinal fluid
	Listeria innocua	GCA_009648575.1						Reference genome
	Listeria ivanovii	GCA_000763515.1						Reference genome
	Listeria welshimeri	GCA_900187315.1						Reference genome
	Listeria grayi	GCA_900638015.1						Reference genome
	Listeria seeligeri	GCA_000027145.1						Reference genome
	Escherichia coli	GCA_000005845.2						Reference genome
	Salmonella enterica	GCA_000006945.2						Reference genome
	Clostridium tetani E88	GCA_000007625.1						Reference genome
	Bacillus anthracis	GCA_000008445.1						Reference genome
	Salmonella bongori	GCA_000439255.1						Reference genome
	Bacillus cereus	GCA_002220285.1						Reference genome
	Corynebacterium diphtheriae	GCA_002843135.1						Reference genome
Total	355							

Table S2. Detailed information on genome size, GC content, number of contigs and N50 of *L. monocytogenes* strains isolated from different regions.

Region	GenBank	Strain names	Genome size (Mbp)	GC content(%)	No. of contigs	N50(Kbp)	GenBank	Strain names	Genome size (Mbp)	GC content(%)	No. of contigs	N50(Kbp)
	GCA_001759995.1	NRRL B-33360	2.8	38.0	15	547.3	GCA_004428295.1	PNUSAL000342	3.0	38.0	28	310.5
	GCA_002131885.1	PNUSAL001135	2.9	38.0	42	167.6	GCA_004428355.1	PNUSAL000942	3.1	38.0	22	348.3
	GCA_002132265.1	PNUSAL000982	3.0	38.0	20	352.9	GCA_004428565.1	PNUSAL000903	2.9	38.0	17	322.7
	GCA_002132505.1	PNUSAL001180	2.9	38.0	17	481.6	GCA_004430015.1	PNUSAL000909	3.0	38.0	19	507.9
	GCA_002132515.1	PNUSAL001177	2.9	38.0	24	262.4	GCA_004430115.1	PNUSAL000914	3.0	38.0	16	510.5
	GCA_002132885.1	PNUSAL001765	2.9	38.0	29	309.5	GCA_004430515.1	PNUSAL000946	2.9	38.0	26	254.6
	GCA_002740915.1	F4244	3.0	38.0	8	738.0	GCA_004433145.1	PNUSAL000970	3.0	38.0	42	185.9
	GCA_003592865.1	PNUSAL004021	3.0	38.0	37	192.7	GCA_004434465.1	PNUSAL000370	3.2	37.5	27	502.0
	GCA_003594045.1	PNUSAL004032	2.9	38.0	63	103.4	GCA_004435305.1	PNUSAL001030	3.1	38.0	15	577.7
	GCA_003606915.1	PNUSAL000115	2.9	38.0	18	590.8	GCA_004444185.1	PNUSAL000886	2.9	38.0	14	324.8
	GCA_003606935.1	PNUSAL000108	3.1	38.0	43	255.1	GCA_004444225.1	PNUSAL001105	3.1	38.0	31	212.2
	GCA_003607175.1	PNUSAL000011	3.0	38.0	19	501.7	GCA_004444265.1	PNUSAL001103	3.0	38.0	21	321.9
	GCA_003607475.1	PNUSAL000550	3.1	38.0	32	476.7	GCA_004446265.1	PNUSAL001183	3.1	38.0	20	727.3
	GCA_003607615.1	PNUSAL000422	3.0	38.0	18	507.3	GCA_004447685.1	PNUSAL001203	3.0	38.0	21	255.4
	GCA_003607895.1	PNUSAL000435	2.9	38.0	19	510.1	GCA_004447965.1	PNUSAL001225	3.0	38.0	18	305.4
	GCA_003607955.1	MOD1_LS149	3.0	38.0	13	476.7	GCA_004448195.1	PNUSAL001238	3.0	38.0	18	437.1
	GCA_003642565.1	PNUSAL004044	2.9	38.0	18	312.8	GCA_004449405.1	PNUSAL000457	3.0	38.0	23	327.5
	GCA_003642725.1	PNUSAL004043	2.9	38.0	15	583.1	GCA_004450185.1	PNUSAL001285	3.0	38.0	20	294.1
	GCA_003678245.1	PNUSAL004067	3.0	38.0	13	550.6	GCA_004450315.1	PNUSAL001297	2.9	38.0	25	288.1
	GCA_003680975.1	PNUSAL004214	2.9	38.0	36	262.9	GCA_004450525.1	PNUSAL001320	3.0	38.0	24	230.4
	GCA_003681735.1	PNUSAL004136	3.0	38.0	21	528.5	GCA_004453285.1	PNUSAL000298	3.0	38.0	12	584.5
	GCA_003682255.1	PNUSAL004115	3.0	38.0	82	103.1	GCA_004453845.1	PNUSAL004287	2.9	38.0	28	186.7
	GCA_003684095.1	J3540	2.9	38.0	17	414.8	GCA_004454895.1	PNUSAL004277	3.0	38.0	118	50.8
	GCA_003685655.1	PNUSAL004249	3.1	38.0	52	116.0	GCA_004455275.1	PNUSAL000488	3.1	38.0	21	362.6
	GCA_003686175.1	PNUSAL004245	3.0	38.0	22	357.1	GCA_004455475.1	PNUSAL004362	3.2	38.0	17	437.0
	GCA_003698345.1	PNUSAL004283	3.0	38.0	25	282.5	GCA_004458345.1	PNUSAL004494	2.9	38.0	33	178.6
	GCA_003737125.1	PNUSAL004463	3.0	38.0	159	38.0	GCA_004461845.1	PNUSAL004562	3.0	38.0	55	115.9
America	GCA_003742045.1	PNUSAL004484	2.9	38.0	35	218.0	GCA_004464625.1	PNUSAL004589	3.0	38.0	22	258.5
(n=223)	GCA_003759905.1	PNUSAL004487	2.9	38.0	36	187.4	GCA_004472985.1	2009L-1366	3.0	38.0	16	408.4
	GCA_004104175.1	PNUSAL000053	2.9	38.0	34	225.6	GCA_004481225.1	PNUSAL001337	3.0	38.0	23	272.9
	GCA_004358645.2	PNUSAL000052	3.0	38.0	12	495.0	GCA_004482825.1	PNUSAL001410	3.1	38.0	25	357.1
	GCA_004383445.1	PNUSAL000931	3.0	38.0	21	476.2	GCA_004483765.1	PNUSAL001388	2.9	38.0	23	437.9
	GCA_004384145.1	PNUSAL001087	3.0	38.0	20	371.7	GCA_004484905.1	PNUSAL001438	3.0	37.5	37	238.7
	GCA_004385245.1	PNUSAL000228	3.0	38.0	14	557.6	GCA_004488045.1	PNUSAL001469	2.9	38.0	18	471.3
	GCA_004386725.1	PNUSAL000480	3.0	38.0	20	424.9	GCA_004489305.1	PNUSAL001407	2.9	38.0	26	292.3
	GCA_004388645.1	PNUSAL001536	3.2	38.0	24	391.3	GCA_004490205.1	PNUSAL001522	2.9	38.0	92	62.0
	GCA_004389185.1	PNUSAL001736	2.9	38.0	15	506.0	GCA_004491665.1	PNUSAL001513	3.1	38.0	27	225.0
	GCA_004390665.1	PNUSAL000044	3.0	38.0	35	325.8	GCA_004491985.1	PNUSAL001548	3.0	38.0	34	176.4
	GCA_004391545.1	PNUSAL000814	3.0	38.0	15	476.3	GCA_004493445.1	PNUSAL001555	2.9	38.0	23	211.7
	GCA_004392565.1	PNUSAL004414	3.0	38.0	65	89.2	GCA_004493545.1	PNUSAL001562	3.0	38.0	81	75.2
	GCA_004393765.1	PNUSAL001281	3.0	38.0	18	540.6	GCA_004496265.1	PNUSAL001644	2.9	38.0	31	192.1
	GCA_004394385.1	PNUSAL004437	2.9	38.0	22	234.5	GCA_004496345.1	PNUSAL001646	3.0	38.0	14	547.5
	GCA_004396975.1	PNUSAL001477	3.0	38.0	84	76.6	GCA_004496985.1	PNUSAL001695	3.0	38.0	21	408.4
	GCA_004407845.1	PNUSAL000339	3.0	38.0	26	365.6	GCA_004498505.1	PNUSAL001725	3.0	38.0	43	124.8
	GCA_004408625.1	PNUSAL004593	3.0	38.0	14	478.3	GCA_004498885.1	PNUSAL001724	2.9	38.0	30	189.9
	GCA_004408685.1	PNUSAL001075	3.0	38.0	28	304.4	GCA_004501185.1	PNUSAL001781	3.0	38.0	16	540.5
	GCA_004409145.1	PNUSAL001132	2.9	38.0	40	156.3	GCA_004508905.1	PNUSAL000041	3.1	37.5	57	215.8
	GCA_004415765.1	PNUSAL001491	2.9	38.0	27	254.0	GCA_004513665.1	PNUSAL000666	3.0	38.0	14	545.9
	GCA_004416525.1	PNUSAL001564	2.9	37.5	38	339.4	GCA_004516245.1	PNUSAL000761	3.1	38.0	63	110.0
	GCA_004417945.1	PNUSAL001766	3.0	38.0	13	520.9	GCA_004516665.1	PNUSAL000180	2.9	38.0	40	159.6
	GCA_004419145.1	PNUSAL000613	3.0	38.0	21	255.6	GCA_004517155.1	PNUSAL000742	3.0	38.0	15	544.2
	GCA_004419175.1	PNUSAL000640	3.0	38.0	37	215.0	GCA_004518945.1	PNUSAL000175	3.0	38.0	23	476.0
	GCA_004419265.1	PNUSAL000030	2.9	38.0	27	501.3	GCA_004519245.1	PNUSAL000176	3.0	38.0	17	542.6
	GCA_004425875.1	PNUSAL000805	3.0	38.0	24	538.3	GCA_004571555.1	PNUSAL002352	2.9	38.0	22	259.0
	GCA_004426535.1	PNUSAL000873	2.8	38.0	25	334.2	GCA_004573095.1	PNUSAL002398	2.9	38.0	15	478.1
	GCA_004427235.1	PNUSAL001589	3.0	38.0	72	102.9	GCA_004573195.1	PNUSAL002357	2.9	38.0	54	103.3
	GCA_002027825.1	MQ130029	3.0	38.0	8	561.5	GCA_003344525.1	356_04	2.9	38.0	38	302.9
	GCA_002027965.1	MQ140030	2.9	38.0	10	501.7	GCA_017659035.1	LmcIH1-4	3.0	38.0	15	477.7
	GCA_002028185.1	MQ150008	3.0	38.0	8	595.9	GCA_019657625.1	LmcINH_2	3.0	38.0	15	436.9
	GCA_002443735.1	CFSAN049290	2.9	38.0	24	565.1	GCA_022375875.1	LmcH67_6	3.0	38.0	15	543.5
	GCA_002443885.1	CFSAN049275	2.9	38.0	29	295.3	GCA_022376055.1	LmcIH1-6	3.0	38.0	18	443.4
	GCA_002443975.1	CFSAN049268	2.9	38.0	21	507.3	GCA_025439835.1	LmcIH1-10	3.0	38.0	36	252.1
	GCA_002444175.1	CFSAN049252	2.9	38.0	25	259.4	GCA_026080615.1	LmcIH1-11	3.0	38.0	24	509.9
	GCA_002444375.1	CFSAN049238	3.0	38.0	30	420.3	GCA_028523205.1	CFSAN061423	2.9	38.0	20	581.7
	GCA_002444445.1	CFSAN049229	3.1	38.0	38	224.8	GCA_028523245.1	CFSAN061395	2.9	38.0	17	629.3
	GCA_002444695.1	CFSAN049283	3.0	38.0	35	244.9	GCA_028528605.1	CFSAN061438	2.9	38.0	15	1500.0
Europe	GCA_002444875.1	CFSAN049251	3.0	38.0	23	435.8	GCA_028528685.1	CFSAN061436	2.9	38.0	20	524.5
(n=91)	GCA_002445155.1	CFSAN049227	2.9	38.0	25	356.8	GCA_028529845.1	CFSAN061396	2.9	38.0	18	459.5
	GCA_003002175.1	CFSAN035163	2.9	38.0	12	579.7	GCA_028530145.1	CFSAN061409	3.0	38.0	22	426.1
	GCA_003002195.1	CFSAN035165	3.1	38.0	26	516.5	GCA_028530405.1	CFSAN061402	3.0	38.0	27	292.4
	GCA_003002215.1	CFSAN035164	3.0	38.0	22	501.5	GCA_028608045.1	CFSAN061457	3.0	38.0	19	525.2
	GCA_003002295.1	CFSAN035168	3.0	38.0	25	477.6	GCA_028750195.1	CFSAN061392	2.9	38.0	25	425.3
	GCA_003002785.1	CFSAN035199	3.0	38.0	42	254.3	GCA_028750785.1	CFSAN061391	2.9	38.0	22	419.4
	GCA_003002835.1	CFSAN035191	3.0	38.0	31	381.7	GCA_029130665.1	LmcZhH_1	3.0	38.0	12	1500.0
	GCA_003045185.1	2016TE2013	2.9	38.0	1	2900.0	GCA_029130685.1	LmcIH1_13	3.0	38.0	14	476.9
	GCA_003045225.1	2016TE3773-1-1	2.9	38.0	1	2900.0	GCA_030730795.1	LmcH67_6	3.0	38.0	21	352.0
	GCA_003045255.1	2016TE3770-1-1	2.9	38.0	1	2900.0	GCA_900017105.1	2842STDY5753991	2.9	38.0	14	355.9
	GCA_003045545.1	2016TE3767-1-1	2.9	38.0	1	2900.0	GCA_900017115.1	2842STDY5753916	3.0	38.0	18	277.1
	GCA_003045605.1	2015TE17781-8	2.9	38.0	1	2900.0	GCA_900017125.1	2842STDY5753966	2.9	38.0	9	1500.0
	GCA_000801575.1	SHL012	2.9	38.0	28	355.4	GCA_021023595.1	201612	3.0	38.0	23	506.6
	GCA_009451485.1	R16.0646	3.0	38.0	13	513.5	GCA_021023705.1	201619	3.1	38.0	66	287.0
	GCA_021021675.1	158	3.0	38.0	17	508.5	GCA_021023975.1	h02	3.1	38.0	72	287.0
Asia	GCA_021021775.1	164	2.9	38.0	16	438.9	GCA_021024015.1	h05	3.0	38.0	14	579.6
(n=29)	GCA_021023205.1	165	2.9	38.0	23	439.3	GCA_021024295.1	h17	2.9	38.0	21	476.8
	GCA_021023255.1	173	3.0	38.0	33	476.7	GCA_021024515.1	h30	3.0	38.0	18	519.1
	GCA_021023435.1	175	3.0	38.0	21	542.5	GCA_021024755.1	h42	2.9	38.0	19	476.8
	GCA_021023575.1	201609	2.9	38.0	12	727.6	GCA_021024815.1	h44	3.0	38.0	17	438.9

Table S2. Detailed information on genome size, GC content, number of contigs and N50 of *L. monocytogenes* strains isolated from different regions.

Region	GenBank	Strain names	Genome size (Mbp)	GC content(%)	No. of contigs	N50(Kbp)	GenBank	Strain names	Genome size (Mbp)	GC content(%)	No. of contigs	N50(Kbp)
America (n=223)	GCA_004573235.1	PNUSAL002349	2.9	38.0	16	408.3	GCA_004639885.1	PNUSAL001606	3.0	38.0	22	361.3
	GCA_004573755.1	TX-ACB1302901	2.9	38.0	22	225.7	GCA_004640585.1	PNUSAL001858	2.9	38.0	79	82.6
	GCA_004574145.1	TX-ACB1301197	3.0	38.0	17	1500.0	GCA_004641445.1	PNUSAL001836	3.0	38.0	21	510.0
	GCA_004574195.1	PNUSAL002340	2.9	38.0	44	129.5	GCA_004641565.1	PNUSAL001847	3.1	38.0	22	477.7
	GCA_004574215.1	PNUSAL002354	2.9	38.0	61	114.4	GCA_004642185.1	PNUSAL001862	3.2	38.0	23	449.2
	GCA_004575375.1	PNUSAL002345	3.0	38.0	15	408.3	GCA_004643125.1	PNUSAL001907	3.0	38.0	20	327.8
	GCA_004576115.1	PNUSAL002443	3.1	37.5	26	476.2	GCA_004644365.1	PNUSAL001928	3.0	38.0	16	476.4
	GCA_004577265.1	PNUSAL002556	2.9	38.0	61	91.2	GCA_004644585.1	PNUSAL001932	3.0	38.0	39	110.7
	GCA_004577695.1	PNUSAL002541	2.9	38.0	65	87.4	GCA_004645285.1	PNUSAL001946	3.1	38.0	29	228.9
	GCA_004581475.1	PNUSAL002608	3.0	38.0	23	243.0	GCA_004646665.1	PNUSAL001807	3.1	38.0	27	254.6
	GCA_004581595.1	PNUSAL002610	3.0	38.0	17	478.1	GCA_004648565.1	PNUSAL001963	2.9	38.0	106	50.1
	GCA_004582375.1	PNUSAL002628	2.9	38.0	20	302.7	GCA_004652125.1	PNUSAL001997	3.0	38.0	47	225.0
	GCA_004582825.1	PNUSAL002640	2.9	38.0	18	408.3	GCA_004652425.1	PNUSAL000810	2.9	38.0	51	142.0
	GCA_004585695.1	PNUSAL002635	2.9	38.0	21	357.1	GCA_004652845.1	PNUSAL002011	2.9	38.0	52	164.0
	GCA_004586235.1	PNUSAL002650	2.9	38.0	13	1500.0	GCA_004654065.1	PNUSAL002041	3.0	38.0	20	492.2
	GCA_004586995.1	PNUSAL002681	3.1	38.0	13	569.0	GCA_004659405.1	PNUSAL002117	3.0	38.0	21	395.0
	GCA_004588095.1	PNUSAL002689	3.1	37.5	34	284.7	GCA_004660625.1	PNUSAL002091	3.0	38.0	66	157.2
	GCA_004588815.1	PNUSAL002728	3.1	38.0	40	177.0	GCA_004660745.1	PNUSAL002093	3.0	38.0	25	294.7
	GCA_004589135.1	PNUSAL002711	2.9	38.0	35	160.0	GCA_004667095.1	PNUSAL002142	3.0	38.0	14	478.2
	GCA_004592455.1	PNUSAL002601	2.9	38.0	29	215.8	GCA_004671105.1	PNUSAL002175	2.9	38.0	16	493.6
	GCA_004592515.1	PNUSAL002761	3.0	38.0	25	218.4	GCA_004673845.1	PNUSAL002204	3.0	38.0	17	584.9
	GCA_004593935.1	PNUSAL002778	3.0	38.0	37	151.4	GCA_004676325.1	PNUSAL002185	2.9	38.0	18	434.8
	GCA_004594105.1	PNUSAL002826	2.9	38.0	22	212.5	GCA_004676785.1	PNUSAL002214	2.9	38.0	97	99.5
	GCA_004594555.1	PNUSAL003049	3.0	38.0	55	255.0	GCA_004677125.1	PNUSAL002206	2.9	38.0	10	545.4
	GCA_004595475.1	PNUSAL003026	2.9	38.0	17	477.6	GCA_004678605.1	PNUSAL002298	2.9	38.0	17	477.4
	GCA_004601475.1	PNUSAL003065	3.0	38.0	63	102.3	GCA_004680305.1	PNUSAL002855	3.0	38.0	55	250.0
	GCA_004601725.1	PNUSAL003105	3.0	38.0	17	408.4	GCA_004681345.1	PNUSAL002857	2.9	38.0	29	238.2
	GCA_004602235.1	PNUSAL003136	2.9	38.0	15	478.2	GCA_004688945.1	PNUSAL002911	3.0	38.0	74	148.4
	GCA_004604395.1	PNUSAL003159	3.0	38.0	18	450.0	GCA_004690945.1	PNUSAL002952	2.9	38.0	17	448.2
	GCA_004606835.1	PNUSAL003206	2.9	38.0	69	85.5	GCA_004691565.1	PNUSAL002941	3.0	38.0	14	546.9
	GCA_004608505.1	PNUSAL003229	3.0	38.0	18	338.6	GCA_004692525.1	PNUSAL002940	3.1	38.0	56	108.0
	GCA_004608755.1	PNUSAL003244	3.0	38.0	19	476.5	GCA_004699265.1	PNUSAL003037	2.9	38.0	17	320.7
	GCA_004608995.1	PNUSAL003269	2.9	38.0	32	184.2	GCA_004700505.1	PNUSAL003788	3.0	38.0	23	259.1
	GCA_004609375.1	PNUSAL003274	3.0	38.0	20	336.9	GCA_004700545.1	PNUSAL003820	3.0	38.0	20	477.3
	GCA_004611395.1	PNUSAL003258	3.1	38.0	27	452.5	GCA_004703565.1	PNUSAL003858	2.9	38.0	33	238.9
	GCA_004611635.1	PNUSAL003315	3.2	38.0	69	107.7	GCA_004705155.1	PNUSAL003939	2.9	38.0	50	139.3
	GCA_004611715.1	PNUSAL003082	2.9	38.0	21	509.8	GCA_004705705.1	PNUSAL003918	3.0	38.0	17	519.7
	GCA_004614395.1	PNUSAL003391	2.9	38.0	113	49.6	GCA_004705725.1	PNUSAL003907	2.8	38.0	55	245.0
	GCA_004616955.1	PNUSAL003464	3.0	38.0	37	148.9	GCA_004706345.1	PNUSAL003942	3.0	38.0	24	446.5
	GCA_004621315.1	PNUSAL003420	3.0	38.0	20	357.1	GCA_004706485.1	PNUSAL003947	3.0	38.0	19	321.2
	GCA_004621555.1	PNUSAL003563	2.9	38.0	19	421.2	GCA_005232775.1	PNUSAL002912	2.9	38.0	14	509.7
	GCA_004622455.1	PNUSAL003557	3.0	38.0	20	447.6	GCA_005233155.1	PNUSAL003618	3.0	38.0	14	310.0
	GCA_004623815.1	PNUSAL003609	3.0	38.0	15	511.4	GCA_005233335.1	PNUSAL002488	2.9	38.0	177	34.6
	GCA_004626575.1	PNUSAL003654	2.9	38.0	50	107.7	GCA_006218645.1	PNUSAL002229	3.0	38.0	12	563.3
	GCA_004629335.1	PNUSAL003759	3.0	38.0	16	478.1	GCA_006218825.1	PNUSAL002247	2.9	38.0	64	114.8
	GCA_004629595.1	PNUSAL003740	2.9	38.0	35	195.2	GCA_006220165.1	PNUSAL002231	3.1	38.0	74	108.2
	GCA_004631435.1	PNUSAL003778	3.2	37.5	26	362.3	GCA_022381435.1	PNUSAL013264	3.0	38.0	12	544.3
	GCA_004631455.1	PNUSAL003752	2.9	38.0	17	477.1	GCA_022692165.1	LIS22-0203	3.0	38.0	140	37.1
	GCA_004632665.1	PNUSAL003317	3.2	38.0	60	100.7	GCA_022693385.1	LIS22-0216	2.9	38.0	105	65.0
	GCA_004632805.1	PNUSAL003291	3.0	38.0	20	299.2	GCA_024687785.1	PNUSAL014745	3.0	38.0	11	561.5
	GCA_004632815.1	PNUSAL003816	2.9	38.0	18	434.8	GCA_025856735.1	PNUSAL015707	3.1	38.0	19	477.1
	GCA_004633395.1	PNUSAL003834	2.9	38.0	214	25.7	GCA_026380935.1	CFSAN090874	3.0	38.0	21	458.9
	GCA_004633935.1	PNUSAL003754	2.9	38.0	18	357.1	GCA_030990985.1	SK2835	2.9	38.0	132	45.2
	GCA_004638005.1	PNUSAL001789	3.0	38.0	21	314.2	GCA_030998675.1	FSL-F2-0605	3.3	38.0	22	448.8
	GCA_004638285.1	PNUSAL001466	2.9	38.0	21	262.3	GCA_030999895.1	FSL-F3-0565	3.1	38.0	25	257.2
	GCA_004638405.1	PNUSAL001795	3.0	38.0	33	177.1						
Europe (n=91)	GCA_900019025.1	2842STDY5753942	2.9	38.0	17	475.7	GCA_900033385.1	2842STDY5753905	3.0	38.0	19	258.4
	GCA_900022055.1	2842STDY5753878	2.9	38.0	9	626.7	GCA_900033395.1	2842STDY5753930	2.9	38.0	13	562.7
	GCA_900022065.1	2842STDY5753889	3.0	38.0	21	286.5	GCA_900033405.1	2842STDY5753945	3.0	38.0	16	616.1
	GCA_900022465.1	2842STDY5753958	3.0	38.0	21	261.2	GCA_900033675.1	2842STDY5753873	3.0	38.0	15	407.4
	GCA_900024455.1	2842STDY5753994	2.9	38.0	19	507.2	GCA_900033695.1	2842STDY5753909	2.9	38.0	14	523.2
	GCA_900024465.1	2842STDY5753911	3.0	38.0	21	403.9	GCA_900034645.1	2842STDY5753892	3.0	38.0	21	255.8
	GCA_900026895.1	2842STDY5753950	2.9	38.0	8	1900.0	GCA_900034655.1	2842STDY5753932	2.9	38.0	12	1500.0
	GCA_900026905.1	2842STDY5753960	3.1	37.5	23	257.6	GCA_900034665.1	2842STDY5753947	3.0	38.0	12	361.7
	GCA_900028125.1	2842STDY5753989	2.9	38.0	14	1700.0	GCA_900034675.1	2842STDY5753962	3.0	38.0	21	286.0
	GCA_900029125.1	2842STDY5753926	3.0	38.0	16	290.3	GCA_900034685.1	2842STDY5753964	3.0	38.0	22	485.6
	GCA_900029865.1	2842STDY5753936	2.9	38.0	16	361.1	GCA_900034695.1	2842STDY5753968	3.0	38.0	18	404.1
	GCA_900031485.1	2842STDY5753859	3.0	38.0	15	505.3	GCA_900034705.1	2842STDY5753970	3.0	38.0	17	412.2
	GCA_900032665.1	2842STDY5753986	3.0	38.0	17	406.4	GCA_900034715.1	2842STDY5753974	2.9	38.0	16	779.7
	GCA_900032675.1	2842STDY5753981	3.0	38.0	15	401.2	GCA_900035185.1	2842STDY5753952	3.2	37.5	35	271.7
	GCA_900032685.1	2842STDY5753854	2.9	38.0	15	520.4	GCA_900036265.1	2842STDY5753938	2.9	38.0	14	740.1
	GCA_900032695.1	2842STDY5753856	2.9	38.0	13	535.8	GCA_900036625.1	2842STDY5753913	3.0	38.0	22	500.8
	GCA_900032705.1	2842STDY5753863	2.9	38.0	12	737.3	GCA_900036735.1	2842STDY5753940	3.0	38.0	11	572.6
	GCA_900032715.1	2842STDY5753883	3.0	38.0	17	278.1	GCA_900037325.1	2842STDY5753923	3.0	38.0	11	338.0
	GCA_900032725.1	2842STDY5753886	3.0	38.0	17	321.4	GCA_900037335.1	2842STDY5753976	3.0	37.5	16	357.3
	GCA_900032735.1	2842STDY5753899	3.0	38.0	15	579.7	GCA_900041415.1	2842STDY5753897	2.9	38.0	11	502.8
	GCA_900032745.1	2842STDY5753903	2.9	38.0	14	442.1	GCA_900043345.1	2842STDY5753870	3.1	38.0	26	278.0
	GCA_900032755.1	2842STDY5753907	3.0	38.0	19	485.6	GCA_900045395.1	2842STDY5753880	3.0	38.0	14	684.4
	GCA_900033375.1	2842STDY5753996	3.0	38.0	10	953.0						
Asia (n=29)	GCA_021025255.1	h66	2.8	38.0	12	1500.0	GCA_030480985.1	LM04	2.9	38.0	11	437.8
	GCA_021025895.1	h91	3.0	38.0	23	405.4	GCA_030481045.1	LM02	2.9	38.0	14	442.3
	GCA_023515845.1	s2020TJ	3.0	38.0	2	3000.0	GCA_030481195.1	LM18	3.0	38.0	9	444.7
	GCA_025681005.1	R19.2864	3.0	38.0	15	562.3	GCA_030481225.1	LM17	2.9	38.0	6	1500.0
	GCA_030480625.1	LM21	3.0	38.0	9	525.4	GCA_030481285.1	LM06	2.9	38.0	14	437.8
	GCA_030480725.1	LM22	2.9	38.0	7	1500.0						
	GCA_030480765.1	LM09	2.8	38.0	7	1500.0						
	GCA_030480935.1	LM19	3.0	38.0	10	444.7						

Table S3. Detailed information on potential target genes of *L. monocytogenes* strains isolated from different regions.

Potential target genes	Name of target ganes	Potential target genes	Name of target ganes	Potential target genes	Name of target ganes	Potential target genes	Name of target ganes
drrA	lmo0979	group_4966	lmo0720	purM	purM	cobD_1	lmo1192
group_3475	lmo0986	ribF_1	lmo0728	pcrB	lmo1760	cbiA	cbiA
group_2643	lmo0987	group_1251	lmo0733	pcrA	pcrA	rhaS_1	lmo1189
group_956	lmo0995	group_3637	lmo0273	ligA	lmo1758	group_3939	lmo1183
dapX	lmo1006	iolG_3	lmo0277	gatA_2	gatA	group_1362	lmo1178
crr	lmo1017	yycH	lmo0289	group_1698	lmo1752	group_3943	lmo1157
cutC_1	lmo1018	yycI	lmo0290	rlmCD_2	lmo1751	cobS	lmo1148
group_1201	lmo1024	group_32878	lmo0292	cbbY	lmo2798	coaE	lmo1563
moeA	lmo1042	mngB_4	lmo2014	bglK_3	lmo2796	mutM	mutM
moaE	lmo1044	group_1894	lmo2013	licR_2	lmo2784	argF	argF
moaB	lmo1048	yurK	lmo2004	group_1304	lmo2781	argJ	argJ
group_424	lmo1050	group_3645	lmo2002	gmuA_1	lmo2780	argC	argC
group_4848	lmo1059	glmS_3	lmo1999	gshAB	lmo2770	ezrA	lmo1594
group_3488	lmo1065	group_3649	lmo1997	ytrB_2	lmo2769	group_3967	lmo1606
group_2661	lmo0401	leuB	leuB	group_9976	lmo2767	group_10180	lmo1621
iolD	lmo0386	group_4985	lmo1963	group_1713	lmo2742	group_10181	lmo1624
iolC	lmo0385	slmA	lmo1962	lrp	lmo2727	trpA	trpA
thlA	lmo1414	ycgT	lmo1961	slyA_2	lmo2726	trpF	trpF
mvaS	lmo1415	yxeB	lmo1959	group_3788	lmo2723	trpC	trpC
group_3504	lmo1416	cmk	cmk	kdpC	kdpC	trpD2	trpD
murB	lmo1420	group_2799	lmo1935	cpdA_2	lmo2642	pabA	trpG
dapA	lmo1435	hepS	lmo1932	sapB	lmo2602	trpE	trpE
lysC_1	lmo1436	aroB	aroB	truA	truA	bcrA_2	lmo1636
asd	lmo1437	hisC	hisC	adhR_2	lmo2593	group_10183	lmo1637
ygaZ	lmo1442	yfeW	lmo1916	yvgN_2	lmo2592	sbcD	lmo1646
dnaG	dnaG	pdeG	lmo1914	hemH	hemH	yheI	lmo1652
group_3522	lmo1487	dgcT	lmo1912	group_2500	lmo2844	rluD_1	lmo0969
aroE_2	lmo1490	group_2801	lmo1911	mvaA	lmo0825	rlhA	lmo0960
group_8648	lmo1495	panB	panB	group_3841	lmo0821	group_5341	lmo0954
trmR	lmo1498	ypmB	lmo1898	group_2290	lmo0665	nanR	lmo0948
rlmI	lmo0581	dnaD	dnaD	group_5184	lmo0666	yycB	lmo0947
group_3530	lmo0585	group_9909	lmo1893	folB	folA	group_3059	lmo0946
group_698	lmo0587	yppE	lmo1890	folP	sul	group_3984	lmo0945
phrB	lmo0588	group_5002	lmo1889	tilS	lmo0219	ltaS1	lmo0927
group_2687	lmo0590	dfrA	lmo1873	group_2296	lmo0212	group_44460	lmo0926
group_2690	lmo0597	ccpN	lmo1865	group_825	lmo0209	ybhS	lmo0925
group_967	lmo0600	group_3673	lmo1862	plcB	plcB	ybhF	lmo0923
group_2692	lmo0603	group_3674	lmo1861	prfA_2	prfA	licC_1	lmo0915
group_3537	lmo0604	msrB	lmo1859	group_10067	lmo0191	group_2388	lmo0911
group_1214	lmo0609	yceM	lmo1858	rnmV	lmo0187	group_1811	lmo0910
slyA_1	lmo0612	yodJ	lmo1855	group_2305	lmo0166	ytrA_1	lmo0909
group_4893	lmo0621	copA	lmo1853	rsbRD_2	lmo0161	group_3988	lmo0900
group_4894	lmo0625	ctpA	lmo1851	yidA_3	lmo0158	alr	dal
group_2489	lmo0626	mntA	lmo1847	dinG_2	lmo0157	hemY	lmo0884
COQ5_2	lmo0637	pyrB	pyrB	group_2308	lmo0156	group_1818	lmo0883
zosA	lmo0641	pyrE	pyrE	znuA_2	lmo0153	group_2396	lmo0879
fsaA	lmo0643	group_3687	lmo1800	group_650	lmo0133	yajO_1	lmo0878
ltaS2	lmo0644	group_1644	lmo1798	group_2314	lmaC	nagB_2	lmo0877
group_9854	lmo0649	hisB	hisB	group_10078	lmo0113	lacF_2	lmo0874
group_3554	lmo0654	hisA	hisA	aes_2	lmo0110	group_4000	lmo0852
pphA	lmo0655	hisI	lmo0562	yodC	lmo0103	malP	lmo2121
group_971	lmo0657	pspB_2	lmo0557	ycnE_1	lmo0102	cdaR	lmo2119
ssuE	lmo2351	dtpT	lmo0555	group_10081	lmo0100	group_5363	lmo2112
tcyM	lmo2347	group_2835	lmo0542	adhR_4	lmo0083	norG	lmo2100
lacC_2	fruB	scmP_1	lmo0538	davD	lmo0913	group_3078	lmo2098
group_4907	lmo2265	MENG	lmo0532	ada	lmo0076	tsaE	lmo2078
group_4908	lmo2262	group_300	lmo0528	prpB	lmo0075	group_4018	lmo2074
celD	lmo2259	mhqA_2	lmo0758	group_2985	lmo2368	group_3082	lmo2071
group_2705	lmo2258	lplJ_2	lmo0764	yhfK	lmo2391	MroQ	lmo2070
group_1219	lmo2246	yhfP	lmo0773	fpuC	lmo2429	ycnJ	lmo2062
adaA	lmo2243	group_9929	lmo0775	group_2328	lmo2435	ycnI	lmo2061
ydiM_2	lmo2238	group_9930	lmo0779	group_1770	lmo2441	yiiM	lmo2060
aroE_3	lmo2236	manX_1	lmo0784	group_10113	lmo2454	group_4022	lmo2059
group_9864	lmo2234	ybaK	lmo0790	group_2991	lmo2462	rsmD	lmo2053
pbpF	lmo2229	ruvA	ruvA	group_2331	lmo2465	coaD	lmo2052
yxlF	lmo2227	group_3716	lmo1528	pgcA_2	lmo2475	tmcAL	lmo2049
fumC	citG	group_3717	lmo1526	galE	galE	mdrP_1	lmo2043
ybdM	lmo1309	bglF_1	lmo1035	group_3220	lmo2479	murE	murE
hflX_2	lmo1296	group_5050	lmo1518	dapH_2	lmo2480	murG	murG
yccU	lmo1285	rcsC	lmo1508	group_5249	lmo2486	divIB	divIB
lacX	lmo1283	essB	lmo0060	group_8689	lmo2487	group_3095	lmo2031
sipV	lmo1270	group_2855	lmo0047	group_2334	lmo2490	group_1097	lmo1400
treA	lmo1254	arcA	lmo0043	group_3000	lmo2492	group_4034	lmo1392
hbp2	lmo2185	degA_1	lmo0031	phoR	phoR	spoIIIE	lmo1386
iolE	lmo2162	ispD	lmo0235	group_3898	lmo2504	fni	lmo1383
group_4936	lmo2142	ispF	lmo0236	degV	lmo2514	buk2	lmo1370
mshD_2	lmo2141	ycaD	lmo1682	yigZ	lmo2516	pta_1	lmo1369
yhaP	lmo2140	menE_1	menE	ywle	lmo2540	tlyA	lmo1366
group_3593	lmo2139	znuA_1	lmo1671	prmC	lmo2542	group_4040	lmo1363
group_44572	lmo1743	group_2222	lmo1667	yslB	lmo1236	xseA	lmo1361
fliY	lmo1738	group_1293	lmo1662	ung_1	lmo1227	group_1838	lmo1352
hcxA	lmo1737	garK_2	lmo2832	group_5295	lmo1225	gcvT	lmo1348
gltC_3	gltC	ycjT	lmo2833	lytG_2	lmo1216	comGC	lmo1345
map	lmo1709	iolG_2	lmo2834	cobQ	cbiP	group_1839	lmo1344
yhfA_1	lmo1704	inlB	inlB	cbiL	cbiL	group_1840	lmo1343
rimJ_1	lmo1698	qorB	lmo0437	cysG	lmo1201	group_3110	lmo1342
recX	lmo1693	group_3177	lmo0451	cbiJ	lmo1200	rsgA_1	lmo1332
mutY	lmo1689	group_2232	lmo0452	cbiG	cbiG		
group_44415	lmo1687	group_5094	lmo0453	cbiT	lmo1196		
group_3631	lmo0704	group_3755	lmo0455	cbiD	cbiD		

Table S4. Detailed information on MLST of *L. monocytogenes* strains isolated from different regions.

Region	GenBank	ST	CC	Lineage	GenBank	ST	CC	Lineage	GenBank	ST	CC	Lineage	GenBank	ST	CC	Lineage
	GCA_001759995.1	ST1187	CC1187	III	GCA_004428295.1	ST5	CC5	I	GCA_004573235.1	ST999	CC554	I	GCA_004639885.1	ST14	CC14	II
	GCA_002131885.1	ST382	CC183	I	GCA_004428355.1	ST5	CC5	I	GCA_004573755.1	ST1	CC1	I	GCA_004640585.1	ST389	CC389	I
	GCA_002132265.1	ST382	CC183	I	GCA_004428565.1	ST1747	CC1747	III	GCA_004574145.1	ST1645	CC19	II	GCA_004641445.1	ST6	CC6	I
	GCA_002132505.1	ST1	CC1	I	GCA_004430015.1	ST5	CC5	I	GCA_004574195.1	ST37	CC37	II	GCA_004641565.1	ST59	CC59	I
	GCA_002132515.1	ST1	CC1	I	GCA_004430115.1	ST230	CC199	II	GCA_004574215.1	ST554	CC554	I	GCA_004642185.1	ST371	CC11	II
	GCA_002132885.1	ST382	CC183	I	GCA_004430515.1	ST1	CC1	I	GCA_004575375.1	ST554	CC554	I	GCA_004643125.1	ST554	CC554	I
	GCA_002740915.1	ST1347	CC6	I	GCA_004433145.1	ST1	CC1	I	GCA_004576115.1	ST1	CC1	I	GCA_004644365.1	ST1842	CC1	I
	GCA_003592865.1	ST7	CC7	II	GCA_004434465.1	ST867	CC867	I	GCA_004577265.1	ST554	CC554	I	GCA_004644585.1	ST554	CC554	I
	GCA_003594045.1	ST768	CC768	II	GCA_004435305.1	ST9	CC9	II	GCA_004577695.1	ST1	CC1	I	GCA_004645285.1	ST6	CC6	I
	GCA_003606915.1	ST834	CC834	II	GCA_004444185.1	ST6	CC6	I	GCA_004581475.1	ST554	CC554	I	GCA_004646665.1	ST6	CC6	I
	GCA_003606935.1	ST2	CC2	I	GCA_004444225.1	ST1	CC1	I	GCA_004581595.1	ST363	CC5	I	GCA_004648565.1	ST1041	CC1041	I
	GCA_003607175.1	ST101	CC101	II	GCA_004444265.1	ST1688	CC1688	I	GCA_004582375.1	ST388	CC388	I	GCA_004652125.1	ST2	CC2	I
	GCA_003607475.1	ST1	CC1	I	GCA_004446265.1	ST155	CC155	II	GCA_004582825.1	ST554	CC554	I	GCA_004652425.1	ST382	CC183	I
	GCA_003607615.1	ST736	CC736	I	GCA_004447685.1	ST1	CC1	I	GCA_004585695.1	ST1	CC1	I	GCA_004652845.1	ST382	CC183	I
	GCA_003607895.1	ST6	CC6	I	GCA_004447965.1	ST1	CC1	I	GCA_004586235.1	ST788	CC7	II	GCA_004654065.1	ST557	CC396	III
	GCA_003607955.1	ST5	CC5	I	GCA_004448195.1	ST7	CC7	II	GCA_004586995.1	ST368	CC368	II	GCA_004659405.1	ST1	CC1	I
	GCA_003642565.1	ST558	CC388	I	GCA_004449405.1	ST554	CC554	I	GCA_004588095.1	ST399	CC14	II	GCA_004660625.1	ST87	CC87	I
	GCA_003642725.1	ST365	CC14	II	GCA_004450185.1	ST217	CC217	I	GCA_004588815.1	ST5	CC5	I	GCA_004660745.1	ST846	CC87	I
	GCA_003678245.1	ST397	CC4	I	GCA_004450315.1	ST554	CC554	I	GCA_004589135.1	ST554	CC554	I	GCA_004667095.1	ST87	CC87	I
	GCA_003680975.1	ST217	CC217	I	GCA_004450525.1	ST639	CC639	I	GCA_004592455.1	ST4	CC4	I	GCA_004671105.1	ST1857	CC1857	III
	GCA_003681735.1	ST369	CC369	II	GCA_004453285.1	ST7	CC7	II	GCA_004592515.1	ST217	CC217	I	GCA_004673845.1	ST29	CC29	II
	GCA_003682255.1	ST2627	CC2627	III	GCA_004453845.1	ST388	CC388	I	GCA_004593935.1	ST2	CC2	I	GCA_004676325.1	ST451	CC11	II
	GCA_003684095.1	ST1508	CC20	II	GCA_004454895.1	ST838	CC838	II	GCA_004594105.1	ST388	CC388	I	GCA_004676785.1	ST382	CC183	I
	GCA_003685655.1	ST7	CC7	II	GCA_004455275.1	ST101	CC101	II	GCA_004594555.1	ST1	CC1	I	GCA_004677125.1	ST1859	CC1859	III
	GCA_003686175.1	ST1	CC1	I	GCA_004455475.1	ST7	CC7	II	GCA_004595475.1	ST219	CC4	I	GCA_004678605.1	ST392	CC392	I
	GCA_003698345.1	ST217	CC217	I	GCA_004458345.1	ST2035	CC4	I	GCA_004601475.1	ST666	CC666	I	GCA_004680305.1	ST5	CC5	I
	GCA_003737125.1	ST6	CC6	I	GCA_004461845.1	ST370	CC366	II	GCA_004601725.1	ST554	CC554	I	GCA_004681345.1	ST1	CC1	I
America	GCA_003742045.1	ST388	CC388	I	GCA_004464625.1	ST663	CC663	I	GCA_004602235.1	ST4	CC4	I	GCA_004688945.1	ST392	CC392	I
(n=223)	GCA_003759905.1	ST296	CC88	I	GCA_004472985.1	ST554	CC554	I	GCA_004604395.1	ST382	CC183	I	GCA_004690945.1	ST219	CC4	I
	GCA_004104175.1	ST6	CC6	I	GCA_004481225.1	ST1331	CC1331	II	GCA_004606835.1	ST1	CC1	I	GCA_004691565.1	ST388	CC388	I
	GCA_004358645.2	ST217	CC217	I	GCA_004482825.1	ST1	CC1	I	GCA_004608505.1	ST382	CC183	I	GCA_004692525.1	ST5	CC5	I
	GCA_004383445.1	ST688	CC688	I	GCA_004483765.1	ST18	CC18	II	GCA_004608755.1	ST217	CC217	I	GCA_004699265.1	ST6	CC6	I
	GCA_004384145.1	ST1	CC1	I	GCA_004484905.1	ST1681	CC4	I	GCA_004608995.1	ST1039	CC2	I	GCA_004700505.1	ST397	CC4	I
	GCA_004385245.1	ST1	CC1	I	GCA_004488045.1	ST1	CC1	I	GCA_004609375.1	ST1	CC1	I	GCA_004700545.1	ST397	CC4	I
	GCA_004386725.1	ST29	CC29	II	GCA_004489305.1	ST155	CC155	II	GCA_004611395.1	ST5	CC5	I	GCA_004703565.1	ST217	CC217	I
	GCA_004388645.1	ST5	CC5	I	GCA_004490205.1	ST554	CC554	I	GCA_004611635.1	ST2	CC2	I	GCA_004705155.1	ST296	CC88	I
	GCA_004389185.1	ST1831	CC1831	III	GCA_004491665.1	ST2	CC2	I	GCA_004611715.1	ST6	CC6	I	GCA_004705705.1	ST230	CC199	II
	GCA_004390665.1	ST791	CC11	II	GCA_004491985.1	ST2	CC2	I	GCA_004614395.1	ST639	CC639	I	GCA_004705725.1	ST554	CC554	I
	GCA_004391545.1	ST1	CC1	I	GCA_004493445.1	ST1809	CC1809	III	GCA_004616955.1	ST101	CC101	II	GCA_004706345.1	ST87	CC87	I
	GCA_004392565.1	ST2	CC2	I	GCA_004493545.1	ST4	CC4	I	GCA_004621315.1	ST1	CC1	I	GCA_004706485.1	ST2	CC2	I
	GCA_004393765.1	ST226	CC226	II	GCA_004496265.1	ST570	CC570	II	GCA_004621555.1	ST796	CC193	II	GCA_005232775.1	ST1917	CC1917	II
	GCA_004394385.1	ST2030	CC2030	III	GCA_004496345.1	ST219	CC4	I	GCA_004622455.1	ST389	CC389	I	GCA_005233155.1	ST382	CC183	I
	GCA_004396975.1	ST217	CC217	I	GCA_004496985.1	ST554	CC554	I	GCA_004623815.1	ST1	CC1	I	GCA_005233335.1	ST2	CC2	I
	GCA_004407845.1	ST87	CC87	I	GCA_004498505.1	ST554	CC554	I	GCA_004626575.1	ST1082	CC1082	II	GCA_006218645.1	ST388	CC388	I
	GCA_004408625.1	ST1	CC1	I	GCA_004498885.1	ST1829	CC1826	III	GCA_004629335.1	ST4	CC4	I	GCA_006218825.1	ST554	CC554	I
	GCA_004408685.1	ST9	CC9	II	GCA_004501185.1	ST226	CC226	II	GCA_004629595.1	ST155	CC155	II	GCA_006220165.1	ST2	CC2	I
	GCA_004409145.1	ST1513	CC1513	III	GCA_004508905.1	ST783	CC4	I	GCA_004631435.1	ST1979	CC1864	II	GCA_022381435.1	ST1046	CC1046	I
	GCA_004415765.1	ST1074	CC1074	I	GCA_004513665.1	ST1519	CC1519	III	GCA_004631455.1	ST308	CC1	I	GCA_022692165.1	ST6	CC6	I
	GCA_004416525.1	ST91	CC14	II	GCA_004516245.1	ST2	CC2	I	GCA_004632665.1	ST1	CC1	I	GCA_022693385.1	ST6	CC6	I
	GCA_004417945.1	ST382	CC183	I	GCA_004516665.1	ST881	CC881	I	GCA_004632805.1	ST554	CC554	I	GCA_024687785.1	ST782	CC2	I
	GCA_004419145.1	ST554	CC554	I	GCA_004517155.1	ST29	CC29	II	GCA_004632815.1	ST451	CC11	II	GCA_025856735.1	ST5	CC5	I
	GCA_004419175.1	ST2	CC2	I	GCA_004518945.1	ST6	CC6	I	GCA_004633395.1	ST35	CC9	II	GCA_026380935.1	ST1062	CC1062	II
	GCA_004419265.1	ST397	CC4	I	GCA_004519245.1	ST792	CC11	II	GCA_004633935.1	ST1	CC1	I	GCA_030990985.1	ST1039	CC2	I
	GCA_004425875.1	ST388	CC388	I	GCA_004571555.1	ST736	CC736	I	GCA_004638005.1	ST1	CC1	I	GCA_030998675.1	ST5	CC5	I
	GCA_004426535.1	ST1743	CC1743	III	GCA_004573095.1	ST4	CC4	I	GCA_004638285.1	ST1	CC1	I	GCA_030999895.1	ST1	CC1	I
	GCA_004427235.1	ST1	CC1	I	GCA_004573195.1	ST378	CC19	II	GCA_004638405.1	ST2	CC2	I				
	GCA_002027825.1	ST1	CC1	I	GCA_003344525.1	ST2	CC2	I	GCA_900019025.1	ST224	CC224	I	GCA_900033385.1	ST6	CC6	I
	GCA_002027965.1	ST4	CC4	I	GCA_017659035.1	ST4	CC4	I	GCA_900022055.1	ST29	CC29	II	GCA_900033395.1	ST6	CC6	I
	GCA_002028185.1	ST431	CC101	II	GCA_019657625.1	ST7	CC7	II	GCA_900022065.1	ST59	CC59	I	GCA_900033405.1	ST101	CC101	II
	GCA_002443735.1	ST29	CC29	II	GCA_022375875.1	ST425	CC90	II	GCA_900022465.1	ST6	CC6	I	GCA_900033675.1	ST616	CC6	I
	GCA_002443885.1	ST1	CC1	I	GCA_022376055.1	ST21	CC21	II	GCA_900024455.1	ST4	CC4	I	GCA_900033695.1	ST1	CC1	I
	GCA_002443975.1	ST6	CC6	I	GCA_025439835.1	ST4	CC4	I	GCA_900024465.1	ST3	CC3	I	GCA_900034645.1	ST2	CC2	I
	GCA_002444175.1	ST1	CC1	I	GCA_026080615.1	ST7	CC7	II	GCA_900026895.1	ST29	CC29	II	GCA_900034655.1	ST391	CC89	II
	GCA_002444375.1	ST38	CC101	II	GCA_028523205.1	ST91	CC14	II	GCA_900026905.1	ST1	CC1	I	GCA_900034665.1	ST613	CC1	I
	GCA_002444445.1	ST2	CC2	I	GCA_028523245.1	ST20	CC20	II	GCA_900028125.1	ST330	CC288	I	GCA_900034675.1	ST6	CC6	I
	GCA_002444695.1	ST14	CC14	II	GCA_028528605.1	ST573	CC573	II	GCA_900029125.1	ST2	CC2	I	GCA_900034685.1	ST6	CC6	I
Europe	GCA_002444875.1	ST9	CC9	II	GCA_028528685.1	ST177	CC177	II	GCA_900029865.1	ST1	CC1	I	GCA_900034695.1	ST6	CC6	I
(n=91)	GCA_002445155.1	ST1	CC1	I	GCA_028529845.1	ST20	CC20	II	GCA_900031485.1	ST412	CC412	II	GCA_900034705.1	ST6	CC6	I
	GCA_003002175.1	ST8	CC8	II	GCA_028530145.1	ST38	CC101	II	GCA_900032665.1	ST6	CC6	I	GCA_900034715.1	ST1	CC1	I
	GCA_003002195.1	ST5	CC5	I	GCA_028530405.1	ST155	CC155	II	GCA_900032675.1	ST6	CC6	I	GCA_900035185.1	ST2	CC2	I
	GCA_003002215.1	ST392	CC392	I	GCA_028608045.1	ST91	CC14	II	GCA_900032685.1	ST6	CC6	I	GCA_900036265.1	ST224	CC224	I
	GCA_003002295.1	ST1	CC1	I	GCA_028750195.1	ST8	CC8	II	GCA_900032695.1	ST1	CC1	I	GCA_900036625.1	ST9	CC9	II
	GCA_003002785.1	ST2	CC2	I	GCA_028750785.1	ST19	CC19	II	GCA_900032705.1	ST1	CC1	I	GCA_900036735.1	ST194	CC315	I
	GCA_003002835.1	ST1	CC1	I	GCA_029130665.1	ST29	CC29	II	GCA_900032715.1	ST2	CC2	I	GCA_900037325.1	ST2	CC2	I
	GCA_003045185.1	ST7	CC7	II	GCA_029130685.1	ST1	CC1	I	GCA_900032725.1	ST2	CC2	I	GCA_900037335.1	ST1	CC1	I
	GCA_003045225.1	ST7	CC7	II	GCA_030730795.1	ST425	CC90	II	GCA_900032735.1	ST8	CC8	II	GCA_900041415.1	ST391	CC89	II
	GCA_003045255.1	ST7	CC7	II	GCA_900017105.1	ST87	CC87	I	GCA_900032745.1	ST8	CC8	II	GCA_900043345.1	ST2	CC2	I
	GCA_003045545.1	ST7	CC7	II	GCA_900017115.1	ST2	CC2	I	GCA_900032755.1	ST6	CC6	I	GCA_900045395.1	ST155	CC155	II
	GCA_003045605.1	ST7	CC7	II	GCA_900017125.1	ST7	CC7	II	GCA_900033375.1	ST120	CC8	II				
	GCA_000801575.1	ST87	CC87	I	GCA_021023595.1	ST59	CC59	I	GCA_021025255.1	ST378	CC19	II	GCA_030480985.1	ST451	CC11	II
	GCA_009451485.1	ST87	CC87	I	GCA_021023705.1	ST8	CC8	II	GCA_021025895.1	ST8	CC8	II	GCA_030481045.1	ST8	CC8	II
	GCA_021021675.1	ST155	CC155	II	GCA_021023975.1	ST8	CC8	II	GCA_023515845.1	ST3	CC3	I	GCA_030481195.1	ST705	CC155	II
Asia	GCA_021021775.1	ST2188	CC8	II	GCA_021024015.1	ST8	CC8	II	GCA_025681005.1	ST87	CC87	I	GCA_030481225.1	ST398	CC19	II
(n=29)	GCA_021023205.1	ST8	CC8	II	GCA_021024295.1	ST224	CC224	I	GCA_030480625.1	ST1	CC1	I	GCA_030481285.1	ST451	CC11	II
	GCA_021023255.1	ST5	CC5	I	GCA_021024515.1	ST3	CC3	I	GCA_030480725.1	ST224	CC224	I				
	GCA_021023435.1	ST121	CC121	II	GCA_021024755.1	ST1	CC1	I	GCA_030480765.1	ST378	CC19	II				
	GCA_021023575.1	ST155	CC155	II	GCA_021024815.1	ST8	CC8	II	GCA_030480935.1	ST705	CC155	II				
